# Deep learning for autosegmentation for radiotherapy treatment planning: State-of-the-art and novel perspectives

**DOI:** 10.1007/s00066-024-02262-2

**Published:** 2024-08-06

**Authors:** Ayhan Can Erdur, Daniel Rusche, Daniel Scholz, Johannes Kiechle, Stefan Fischer, Óscar Llorián-Salvador, Josef A. Buchner, Mai Q. Nguyen, Lucas Etzel, Jonas Weidner, Marie-Christin Metz, Benedikt Wiestler, Julia Schnabel, Daniel Rueckert, Stephanie E. Combs, Jan C. Peeken

**Affiliations:** 1 Institute for Artificial Intelligence and Informatics in Medicine, Klinikum rechts der Isar, Technical University of Munich, Ismaninger Str., 81675 Munich, Bavaria Germany; 2 Department of Radiation Oncology, TUM School of Medicine and Health, Klinikum rechts der Isar, Technical University of Munich, Ismaninger Str., 81675 Munich, Bavaria Germany; 3 Department of Neuroradiology, TUM School of Medicine and Health, Klinikum rechts der Isar, Technical University of Munich, Ismaninger Str., 81675 Munich, Bavaria Germany; 4 Institute for Computational Imaging and AI in Medicine, Technical University of Munich, Lichtenberg Str. 2a, 85748 Garching, Bavaria Germany; 5 Munich Center for Machine Learning (MCML), Technical University of Munich, Arcisstraße 21, 80333 Munich, Bavaria Germany; 6 Konrad Zuse School of Excellence in Reliable AI (relAI), Technical University of Munich, Walther-von-Dyck-Straße 10, 85748 Garching, Bavaria Germany; 7 Department for Bioinformatics and Computational Biology – i12, Technical University of Munich, Boltzmannstraße 3, 85748 Garching, Bavaria Germany; 8 Institute of Organismic and Molecular Evolution, Johannes Gutenberg University Mainz (JGU), Hüsch-Weg 15, 55128 Mainz, Rhineland-Palatinate Germany; 9 Institute of Radiation Medicine (IRM), Helmholtz Zentrum, Ingolstädter Landstraße 1, 85764 Oberschleißheim, Bavaria Germany; 10 Partner Site Munich, German Consortium for Translational Cancer Research (DKTK), Munich, Bavaria Germany; 11 Institute of Machine Learning in Biomedical Imaging, Helmholtz Munich, Ingolstädter Landstraße 1, 85764 Neuherberg, Bavaria Germany; 12 School of Biomedical Engineering & Imaging Sciences, King’s College London, Strand, WC2R 2LS London, London UK; 13 Faculty of Engineering, Department of Computing, Imperial College London, Exhibition Rd, SW7 2BX London, London UK

**Keywords:** Deep learning, Automatic segmentation, Radiotherapy planning, Radiation oncology

## Abstract

The rapid development of artificial intelligence (AI) has gained importance, with many tools already entering our daily lives. The medical field of radiation oncology is also subject to this development, with AI entering all steps of the patient journey. In this review article, we summarize contemporary AI techniques and explore the clinical applications of AI-based automated segmentation models in radiotherapy planning, focusing on delineation of organs at risk (OARs), the gross tumor volume (GTV), and the clinical target volume (CTV). Emphasizing the need for precise and individualized plans, we review various commercial and freeware segmentation tools and also state-of-the-art approaches. Through our own findings and based on the literature, we demonstrate improved efficiency and consistency as well as time savings in different clinical scenarios. Despite challenges in clinical implementation such as domain shifts, the potential benefits for personalized treatment planning are substantial. The integration of mathematical tumor growth models and AI-based tumor detection further enhances the possibilities for refining target volumes. As advancements continue, the prospect of one-stop-shop segmentation and radiotherapy planning represents an exciting frontier in radiotherapy, potentially enabling fast treatment with enhanced precision and individualization.

## Introduction

The rapid development of artificial intelligence (AI) techniques poses great promise for the clinical discipline of radiation oncology. The radiotherapy planning process constitutes an ideal candidate for automation and enrichment by AI techniques due to its largely computational basis and relation to medical imaging.

A key component of the radiation oncology workflow constitutes the graphic distinction of volumes destined to be irradiated from organs at risk (OARs) with specific dose constraints. This process comprises three-dimensional definition of OARs, the gross tumor volume (GTV), the clinical target volume (CTV), and, finally, the planning target volume (PTV). All can be actively contoured or at least supported by the application of AI techniques.

Initially, autosegmentation techniques largely relied on conventional methods, including intensity analysis, shape modeling, and atlas-based techniques. These traditional methods, though innovative at their time, faced challenges in terms of accuracy, efficiency, and adaptability, especially when dealing with complex anatomical variations and diverse cancer types [1].

With the advent of deep learning models, especially convolutional neural networks (CNNs), there has been a paradigm shift in autosegmentation approaches. CNNs, as multilayer feed-forward neural networks, have the capability to extract low-level image features through early hidden layers and progressively learn higher-level features, leading to more accurate and reliable segmentation outcomes [2]. This advancement was crucial for effective radiotherapy planning, where precision is paramount [3].

Deep learning-based models have shown significant promise in various aspects of radiotherapy planning, including for the segmentation of OARs and CTVs. These models are typically developed using retrospective peer-reviewed treatment contours and have been validated to approximate clinical contours for most OARs [4]. However, it is observed that structures with more variability tend to be less accurately segmented, indicating a need for more extensive training data or novel training approaches to improve performance [5].

The integration of these deep learning models into clinical workflows has been a notable advancement. Recent studies highlight the practical application of these models in clinical workflows for various cancer types, including central nervous system, head and neck, prostate, and rectal cancers [6, 7]. This integration showcased the potential of deep learning models to improve the efficiency and consistency of radiotherapy treatment planning, while also emphasizing the importance of continuous refinement and validation.

In this review, we aim to give a technical introduction to the historical development of contouring and current state-of-the-art AI techniques that constitute the basis for contemporary and future models. Moreover, we summarize the evidence for clinical application of autocontouring algorithms. Finally, we provide an outlook on the future of AI for personalized target volume definition.

## Technical basis of autocontouring

### Historic development

The evolution of autosegmentation in radiotherapy treatment planning has been significant, transitioning from traditional methods to advanced deep learning-based approaches. The early methods were primarily based on intensity analysis, shape modeling, and atlas-based approaches [8].

Intensity analysis models rely on the intensity values within the images to differentiate between various tissues. The segmentation is based on the premise that different tissues will have distinct intensity signatures in the imaging modalities used in radiotherapy planning [9, 10]. Though rudimentary, this method laid the groundwork for more advanced segmentation techniques.

Shape modeling incorporates the use of statistical shape models or statistical appearance models to anatomically define plausible shapes. These models were designed to represent the typical anatomy of the structures of interest, aiding in the segmentation process by providing a reference shape against which patient images could be compared [11, 12]. This approach was limited by the lack of flexibility to adapt to significant variations in patient anatomy.

Atlas-based models represented a significant advancement in segmentation. These models use a database of previously delineated OARs and CTVs (an *atlas*) to guide the segmentation process [13, 14]. By comparing a new patient’s images to these atlases, the system can approximate the delineation of the regions of interest. The accuracy of atlas-based segmentation models largely depends on the similarity between the patient’s anatomy and the atlas images. To further improve the performance of these models, over time, they evolved to incorporate multiple atlases and more sophisticated algorithms for atlas selection and adaptation, thereby improving their accuracy and efficiency in segmenting complex anatomical structures [15, 16]. This approach, however, still requires subsequent manual editing to achieve clinical accuracy, since the base accuracy is limited by the diversity of the atlases and may differ greatly from patient to patient [17]. However, many older autocontouring tools in clinically approved softwares are based on this technique.

The introduction of CNNs marked a pivotal point in the evolution of image delineation, moving away from tools that usually relied on manual editing to finish segmentation or achieve clinical accuracy and toward a more autonomous segmentation approach. A step beyond intensity analysis, shape modeling, and atlas-based methods, CNNs leverage deep learning to automatically learn features from imaging data [5]. These models can handle a wider variety of complex anatomical structures and variations among patients, thus improving the accuracy and efficiency of segmenting OARs and CTVs. The higher performance of CNNs lies in their ability to extract hierarchical features from medical images [18] through layers of learned convolutional filters.

### Established deep learning techniques

Deep learning research for medical imaging is mainly inspired by computer vision in the natural domain and adopts techniques for medical tasks. The main differences are scarce training data, higher numbers of modalities, the 3D nature of tomographic imaging, and so-called domain shifts between medical centers in terms of their medical practices and image acquisition with varying protocols and scanners.

**Fig. 1:**
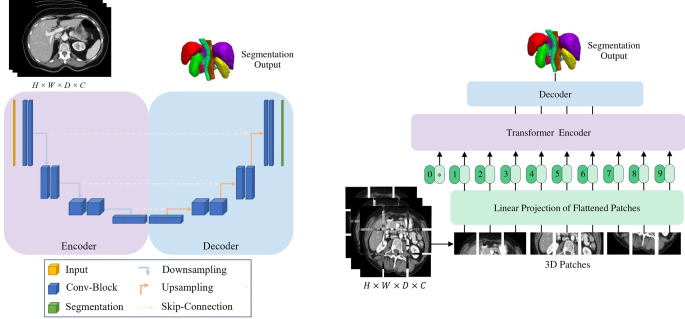
Model overview of fully convolutional U‑Net (**a**) and hybrid convolutional neural network (CNN)–transformer UNetR (**b**). The difference between the architectures is that the CNN encoder in U‑Net is replaced by a transformer-based encoder. For the transformer, the input image has to split into patches and is projected into an embedding space. The U‑Net-specific skip connections between encoder and decoder are also present in UNetR. The figure was inspired by and adopted from [19] and [28]

Common deep learning segmentation approaches are fully convolutional networks of the U‑Net or ResNet variants [19, 20]. The U‑Net architecture is by far the most widely applied architecture and consists of contracting and expanding parts, namely encoder and decoder, resulting in the U‑shaped architecture. Feature representations in the encoder are copied to the decoder via skip connections to enhance spatial details of the segmentation, as shown in Fig. 1. The fully autoconfiguring nnU-Net framework is often used as a baseline and extended to problem-specific needs [21–23].

Since transformer architectures were adopted by the vision domain [24], their use in the medical domain has also been growing. Transformers were originally developed to capture long-range dependencies in natural language processing and built on the core concept of self-attention [25]. In comparison to transformers, CNNs have a better inductive bias for image processing, including translation invariance, partial scale invariance, and multi-scale processing blocks [26]. On the contrary, transformers need to learn all of these image-specific concepts from training samples, such that the high demand for training data also limits their application in the medical domain. Thus, hybrid CNN–transformer architectures are being introduced. These mostly follow a U-Net hierarchical encoder–decoder structure like Swin-UNetR, UNetR, and nnFormer [27–29]. In Fig. 1, the U‑Net and the UNetR architectures are depicted.

Another important aspect of training deep learning architectures entails self-supervised pretraining tasks. Especially for transformer architectures in a low-data regime scenario, this form of pretraining is highly beneficial [27]. Pretraining techniques can include different approaches to learning appearances via non-linear transformation, textures via local pixel shuffling, contexts via out-painting and in-painting as in Model Genesis [30], and also contrastive learning as for the Swin-UNetR [27].

One of the first segmentation models in medical imaging to integrate vision–language encoders was the CLIP-Driven Universal Model [31]. The main idea is to use the pretrained vision–language CLIP encoder [32]. The authors claim that the CLIP embedding captures anatomical relationships, resulting in a more meaningful embedding space than one-hot encoding.

Foundation models, which are trained on vast amounts of data and are capable of segmenting any object, are gaining popularity. Most recently, the UniverSeg model was published and can perform numerous medical contouring tasks in a meta-learning fashion [33]. It is a few-shot model that achieve downstream inference based on a number of example image–annotation pairs with the help of its newly introduced cross-blocks and does not require any further training. This enables clinical researchers, who often lack the resources and expertise to train a deep learning model, to apply autosegmentation to their specific problems.

### Interactive deep learning-based segmentation—hybrid approaches

In many cases, the autosegmentation results require further editing by experts. The most straightforward hybrid approach entails an expert manually perfecting the predicted contours, which is also the current standard for clinical-grade algorithms. As the required effort does not bespeak large datasets, further methods are being explored.

Traditional segmentation methods guided by explicit anatomical descriptors such as shape priors, appearances, motion, and context information differ from deep learning approaches that implicitly learn anatomical features for segmentation. A hybrid model combining both strengths is anticipated to enhance segmentation performance. This fusion could leverage data-driven methods for parameterization and regularization in model-driven approaches and, reciprocally, model-driven methods could contribute to data-driven approaches by assisting in data augmentation, pre- and post-processing, loss function, and regularization [34, 35]. For instance, Ding et al. introduced an automatic contour refinement process employing model-driven methods to enhance deep learning-based segmentation in abdominal magnetic resonance imaging (MRI) scans by implementing a level-set-based active contour model [36]. Yang et al. proposed a model-data-driven hybrid-fusion network, integrating a traditional curvature regularization loss function into the training process to improve segmentation edge smoothness [37].

As further automation for contour refinement, an AI model can learn the editing process and strategy via reinforcement learning (RL) [38]. Liao et al. [39] introduced a multi-agent RL model for iterative refinement of 3D MRI segmentation in a dynamic and interactive fashion. Each voxel is treated as an individual agent, and its state is characterized by four components: voxel value, the previously predicted probability for a specific class, and two hint maps (object hint map and background hint map). These hint maps are generated based on the expert’s mouse clicks, indicating locations of segmentation errors. This sequence of actions iteratively adjusts the segmentation probability to a precise level. Ma et al. expanded the expert interaction to a super-voxel clicking strategy and proposed a boundary-aware reward function [40]. The approach demonstrated increased robustness and accuracy, with a reduced number of interactions across four benchmark datasets.

However, RL-based contour refinement poses several challenges: designing an interaction pattern that effectively and precisely conveys the experts’ intentions is difficult, modeling the dynamic interaction process as appropriate reward functions is also challenging, and, ultimately, the search in the broad action space is time inefficient.

### Explainability and uncertainty within contemporary methods

Within the realm of deep learning, explainability refers to a technique that facilitates understanding an AI algorithm and its decision-making process. This is achieved by disclosing its reasoning, functioning, or behavior in human-understandable terms [41–43]. Particularly in critical tasks like radiotherapy treatment planning, where patient safety is at stake, explainability constitutes a key factor, as the precision and reliability of the predicted segmentation masks directly affect the patient’s health condition. Additionally, explainability can serve the purpose of demonstrating that the model’s decisions align with the clinician’s expertise [44].

Broadly speaking, explainable AI methods can be categorized into visual and non-visual approaches, both resulting in attribution maps (i.e., heatmaps) indicating the contribution of the spatial input features to the activation of the output segmentation. Notably, the visual approach is the more commonly adopted choice in medical image analysis, predominantly selected for its inherent ease of understanding and interpretability [44].

**Fig. 2:**
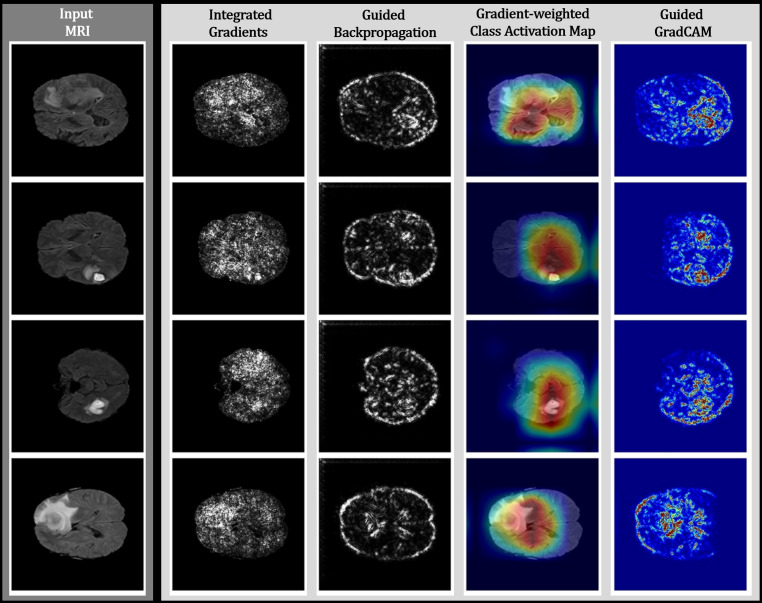
Qualitative results of different visual explainable artificial intelligence methods for high-grade glioma cases of the BraTS dataset, including integrated gradients (IG), guided backpropagation (GBP), gradient-weighted class activation mappings (Grad-CAM), and guided Grad-CAM. Contributing features are highlighted in *white* for IG and GBP, whereas *red* regions correspond to a high score in Grad-CAM and guided Grad-CAM. The figure is partially adopted from [53]

On the one hand, visual approaches can be further classified into perturbation-based methods, which analyze the effect of altering the input features while measuring the deviation from the initial prediction to assess the significance of the input features (i.e., pixel/voxel). Prominent representatives are methods such as occlusion [45], local interpretable model-agnostic explanations [46], Shapley additive explanations [47], or randomized input sampling for an explanation of black-box models [48]. On the other hand, the second facet of visual approaches involves backpropagation-based methods. Therein, one or more forward passes are performed, and partial derivatives are calculated within the neural network during the backpropagation stage to estimate the impact of gradients, weights, and activations, referring to saliency maps, relevance maps, and class activation maps, respectively [49]. Noteworthy examples include integrated gradients (IG) [50], guided backpropagation (GBP) [51], deconvolution networks (DeconvNet) [45], gradient-weighted class activation mappings (Grad-CAM) [52], or guided Grad-CAM [52] (Fig. 2).

Non-visual approaches, including textual, auxiliary, and case-based techniques, constitute another component of explainable AI methods [54]. While often deemed independent of explainability, Poceviciute et al. regard uncertainty quantification as an integral facet of the explainable AI landscape [55]. Two primary types of uncertainty exist [56]. Firstly, epistemic uncertainty refers to the model’s uncertainty caused by a lack of knowledge about the underlying data distribution. However, this may be reduced on the basis of additional data. Prominent approaches to estimating epistemic uncertainty include Bayesian neural networks [57, 58], deep model ensembles [59], and Bayesian approximation using Monte Carlo dropout methods [60, 61]. Secondly, aleatoric uncertainty originates from the underlying data-generation process and includes noise, measurement errors, or geometric transformations [62]. Approaches to estimating aleatoric uncertainty include test-time augmentation [63] and learned loss attenuation [64]. Although tempting, interpreting softmax outputs as a measure of uncertainty tends to be insufficient, as neural networks are often too confident in their predictions [65]. However, if correctly calibrated, predicted probabilities can be interpreted as confidence measures [65]. In summary, deploying a deep learning model for medical image tumor segmentation necessitates a comprehensive understanding of its inner workings to detect errors and biases, thereby facilitating effective clinical integration.

### Generative models

Generative models play a pivotal role in advancing computer vision tasks, with applications ranging from image synthesis to semantic segmentation. Two prominent approaches in the realm of generative modeling are generative adversarial networks (GANs) [66] and denoising diffusion models [67–69]. GANs consist of a generator creating realistic data and a discriminator distinguishing between real and generated samples. Both players increasingly improve until the generator synthesizes realistic images. On the other hand, denoising diffusion models learn to synthesize data by learning to reverse the process of adding randomly distributed noise on top of the original image in an iterative fashion. Both of these methods are often used to enable semantic segmentation through synthetic labeled data and are particularly useful when only small amounts of labeled data are available, as is often the case in medical imaging.

The most prominent approach of generative modeling for semantic segmentation in the natural domain is DatasetGAN [70]. For the first time, this model generates not only synthetic images but also the ground truth, i.e., semantic segmentation maps. Thereby, from a handful labels, a large dataset can be generated, which in turn can be used to train downstream segmentation networks. Similar work by Li et al. [71] shows that employing generative modeling for semantic segmentation facilitates improved out-of-domain semantic segmentation. An example of this within the medical context could be segmentation of computed tomography (CT) modalities by a model trained on MRI scans.

**Fig. 3:**
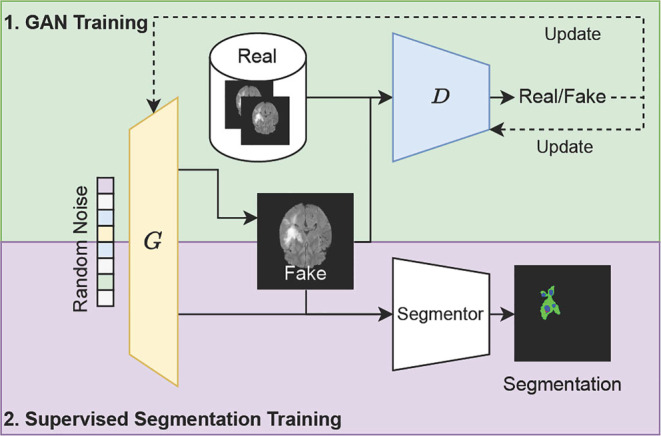
Simplified schematic of DatasetGAN [70], a GAN capable of synthesizing images with the corresponding semantic segmentation label for artificial dataset generation

Abdal et al. [72] find that the established StyleGAN [73] holds properties that can be easily leveraged to obtain fore- and background segmentation maps without additional annotations.

It is often desirable to not only have fixed class labels in semantic segmentation but to also have arbitrary segment classes through an open vocabulary. Xu et al. enable such open-vocabulary semantic segmentation by utilizing the existing clusters in denoising diffusion model representations [74], which could be useful in medical scenarios where radiologists want to freely describe the desired areas in an image.

#### In medical imaging

These works in natural computer vision often serve as foundations for medical imaging applications of generative models for semantic segmentation. Rosnati et al. [75] further analyze SemanticGAN [71] and show that learning the image and label maps jointly, compared to only learning the label maps, is vital for medical images.

Finally, GANs are also used to simply improve existing semantic segmentation approaches. Multiple works [76–78] find improved segmentation performance when adding an adversarial loss to their segmentation pipeline. Xue et al. [79] further improve upon these concepts with novel multi-scale loss functions.

### Metrics

When developing and testing automatic segmentation, it is necessary to study the performance and to quantify it, so that the optimal models can be defined. Most studies employ a combination of qualitative and quantitative metrics to assess autosegmented contours based on reference ones. The quantitative metrics that measure the agreement among contours can be divided into two categories: volume-based and distance-based metrics.

Volume-based methods typically measure the overlapping portions of two shapes as ratios. The used denominator varies among metrics, e.g., the volumes of shapes summed (*Dice similarity coefficient*, DSC) [80], the volume of the union of shapes (*Jaccard index*) [80], the autosegmented contour (*precision/positive predictive value*) [81], or the ground-truth volume (*recall/sensitivity/true-positive rate*) [81]. As a remark, these metrics are not completely independent; e.g., DSC can be derived from the others. Therefore, DSC is often used alone as a single all-comprehensive metric for volume-based evaluation.

Distance-based evaluators quantify the spatial separation between two structures, e.g., the numeric distance between the predicted and reference contours (*Hausdorff distance*, HD [82]; and *surface DSC* [83]) specifically designed for radiotherapy-relevant evaluations.

Further methods such as subjective inspection, efficiency, and dosimetric metrics are also used. Table 1 summarizes the most common means of evaluating medical autosegmentation.

**Table 1:** Metrics to assess autosegmentation performance in the medical domain. Source for images [8]

Evaluation	Example	Advantage	Drawback
*Volume-based metrics*	– Dice similarity coefficient (DSC) [80] – Jaccard conformity index [80] – Precision/positive predictive value [81] – Recall/sensitivity/true-positive rate [81]	– Numerical scores – Easy to compute – Objective inspection – Capture volume/contour overlap	– Biased on larger instances – Insensitive to contour deviation for large structures – Highly sensitive to deviation for small structures
*Distance-based metrics*	– Hausdorff distance (HD) [82] – Mean surface distance (MSD) [84] – Surface DSC [83] – 95th percentile Hausdorff distance (HD95) [84]	– Numerical scores – Objective inspection – Captures discrepancy in distance	– Requires pre-specified tolerance thresholds – Single metric cannot capture the overall contour discrepancy
*Subjective evaluation*	– Clinician review – Multi-clinician Turing test	– Full-picture evaluation – Shown to predict outcomes – Clinically relevant	– Observer and experience dependant – Time consuming
*Efficiency metrics*	– Saved time [85, 86]	– Easy to quantify – Reflects clinical acceptability	– Observer dependant (for baseline) – Does not reflect segmentation quality alone
*Dosimetric metrics*	– Dose–volume histogram metrics [87] – Isodose lines	– Objective – Clinically relevant	– Requires treatment planning – Large deviations in contours could be accepted

#### Limitations of different metrics

First of all, volume- and distance-based metrics rely on ground truth contours being available and of perfect quality. However, human errors, bias, and inter-observer variations exist even among expert clinicians [88–90]. Hence, comparing metrics across various studies may not be straightforward without understanding the quality of the respective reference contours. This also underscores that a faultless match on metric scores may not always imply perfect real-world performance [91].

Given the limitations inherent to each validation metric, utilizing a combination of evaluation approaches is generally advisable. Nevertheless, a broad combination of metrics may still not exhibit a strong correlation with clinical acceptability [92]. Different approaches and standards may be relevant depending on the nature of the contoured organs. A recent study employing 27 volume- and distance-based accuracy metrics highlighted that the limitations of certain metrics could be offset by others [93].

In some works, human experts are consulted to determine the accuracy and clinical usability of autosegmented contours. This is best performed as a Turing test by multiple clinicians, i.e., without knowing how the contour was generated, in order to alleviate human bias. A recent study has shown that autosegmented contours were preferred over human annotations by evaluating experts [94]. However, naturally, this approach requires much more time and human effort.

## Clinical application

Along the planning process for radiotherapy, almost every step is prone to inter- and even intra-rater variability and is time consuming for the individual physician [95, 96]. The latter is especially strenuous when considering OAR delineation, as a multitude of objects have to be segmented exactly [96–99]. Determining the ground truth GTV became manageable with advancements in imaging modalities, thus providing physicians with information about anatomical constraints (CT, MRI) and physiological processes (positron emission tomography [PET]). Still, clinical segmentations show inconsistencies that impede not only general quality assessment but also a crucial multi-institutional comparison of treatment strategies for research and development [100, 101]. Automated segmentation models may improve the clinical workflow by increasing consistency and efficiency. The current data on clinical applications of autosegmenting tools will be discussed in the following.

### Organs at risk

Radiotherapy plans need to be precise as well as individual. Furthermore, each organ has different dose constraints. Hence, for every patient, the OARs need to be delineated individually.

Various commercially available programs for automatic segmentation exist, such as AI-Rad Companion (Siemens Healthineers, Erlangen, Germany) [102], INTContour (CarinaAI Medical, Lexington, KY, USA) [103, 104], Limbus AI (Limbus AI Inc., Regina, SK, Canada), Mirada Deep Learning Contouring (Mirada Medical Ltd., Oxford, UK) [105], MVision (MVision AI, Helsinki, Finland) [106], Radformation AutoContour (RADformation, New York, NY, USA), Raystation (RaySearch Laboratories, Stockholm, Sweden) [107], or TheraPanacea (Paris, France) [108] (non-exclusive list). The majority of these applications use a U-Net-based architecture for segmentation [109]. Totalsegmentator offers a freeware based on nnU-Net that can be used for OAR segmentation for research projects. It can perform automatic segmentation of 117 classes in CT images. However, it lacks the specifications for radiotherapy and integration into existing systems necessary for clinical application and has no medical product certification [110].

Concerning the quality of the automatic segmentations for OAR, the commercial models perform better or at least to the same level as atlas- or model-based methods at nearly any treatment site. Chen et al. could show that deep learning-based autosegmentation of the masticatory muscles outperforms atlas-based segmentations with a DSC of up to $0.89\pm 0.02$ vs. $0.85\pm 0.04$ and no qualitative differences when comparing dosimetric endpoints to manually segmented contours [85]. Working on thoracic CT scans, a CNN even outperformed physicians, with an average DSC ranging from 0.726 to 0.979, and reduced the editing time to 7.5 min for each patient [103]. Lustbert et al. could show that with deep learning OAR segmentation for non-small cell lung cancer (NSCLC) patients, the median time saved was 10 min compared to manual contouring. Again, the deep learning approach beats the atlas-based segmentation by a median of 7.8 min [105]. The trend of deep learning models superseding atlas-based approaches was also confirmed in a review by Vrtovec et al. for cancers of the head and neck [15]. Even though segmentations of small (e.g., optic nerve, glottis) or mobile organs (e.g., stomach) still needed manual adjustments, Strolin et al. observed a significant reduction in contouring time. Moreover, even after manual adjustment, the deep learning-based segmentations still showed increased consistency compared to fully manual delineations, adding clinical value to this method in addition to speeding up workflows [111]. However, for successful application of autosegmentation models to a local dataset, fine-tuning with transfer learning may be necessary due to differences in image acquisition techniques [104].

During inference, an autosegmentation model will provide equal and robust results every time. In contrast to CTV or GTV delineations, the guidelines for OAR segmentations are not likely to change significantly, allowing for the models to be used in the long term. Moreover, autosegmentation can be used outside the clinical planning workflow for educational purposes. It enables residents or trainees to not only affirm their anatomical knowledge but can also pinpoint guideline deviations later on [112–114].

### Gross tumor volume segmentation

Serving as the basis for CTV and PTV definition, the GTV delineation represents a time-consuming and crucial step in the radiation treatment planning process. Depending on the respective cancerous entity, there is a wide variation in case-specific tumor burden (e.g., primary, lymph node, or metastatic lesions) and possible locations, which thus requires expert assessment of the available clinical data and imaging modalities. An exact voxel-wise differentiation of affected tissue from the surrounding normal tissue is not always possible and often depends on the expert knowledge of the radiation oncologist. The optimal true segmentation is subject to inter-rater differences but is of high importance to ensure adequate dose coverage of the PTV constructed from the GTV, thus ensuring a minimal risk of treatment failure. In turn, if the GTV extends beyond the tumor site, subsequent dose escalation in the adjacent healthy tissue can incur unnecessary treatment-related toxicity [115].

**Fig. 4:**
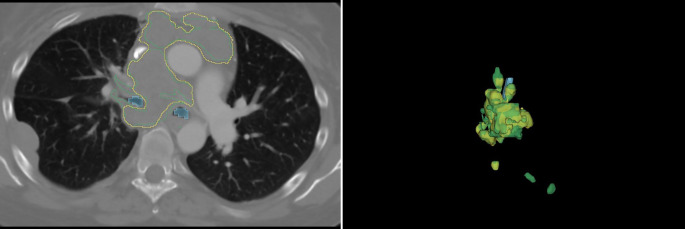
**Automatic segmentation for suspect mediastinal lymph nodes.** Set of ground truth annotations (*green*) and model predictions (*yellow*). For improved visualization the trachea is shown in *blue*. Dice similarity coefficient 0.663. Provided by Fischer et al. [117]

The advent of deep learning has led to the development of semi- or fully automatic algorithms, thus allowing for faster GTV delineation that may be more robust to variations between different raters. Primakov et al. managed to generate a model that reaches a DSC of 0.82 and an HD95 of 9.43 for the automatic segmentation of the NSCLC GTV in thoracic CT scans. It was accompanied by a considerable time reduction from $172.19\pm 158.99$ s per patient for manual segmentations to $2.78\pm 0.44$ s with the automated method. Among all experts, the median DSC for intra-observer variability was 0.88 (interquartile range, $\text{IQR}=0.12$), whereas automated segmentations exhibited 100% reproducibility. Qualitatively, the automatic segmentations were preferred by radiologists and radiation oncologists in 59% [116]. Another automatic segmentation for the thoracic region was devised by Fischer et al. Using the LNQ2023 dataset of contrast-enhanced thoracic CT images, the latter authors achieved an overall mean DSC of 0.663 with an nnU-Net extended by a preprocessing specialized on weakly supervised annotations (Fig. 4) [117].

Another approach relying solely on CT scans was developed by Skylar et al. for GTV segmentation in head and neck radiotherapy. With a nnU-Net they accomplished a median DSC from 0.6 to 0.7, surface Dice from 0.30 to 0.56, and HD95 from 14.7 to 19.7 mm across five different approaches. Though they outperformed autocontouring based on multiple modalities, delineations need to undergo edits before clinical use, thus demonstrating the challenging nature of head and neck primary tumor delineation [118]. Liao et al. validated their MRI-based semi-supervised learning network for GTV segmentation in nasopharyngeal carcinoma at a DSC of 0.83 for the nasopharynx and 0.80 for nodes. Additionally, the efficiency in delineating could be improved by over 60% [119].

For GTV segmentation of large brain metastases, Buchner et al. developed a 3D-U-Net on MRI scans with a final mean overall DSC of $0.92\pm 0.08$ [120]. Again, this performance was only slightly worse compared to the intra- and inter-observer variabilities, with DSCs of 0.95 and 0.94, respectively. Further on, they performed an ablation study to reduce the model size and number of required MRI sequences to one sequence only [121]. Based on a multi-modality (CT and MRI) approach, Tian et al. implemented a deep learning model for glioblastoma autosegmentation. It performed with a DSC of 0.94 and HD95 of 2.07 mm [122].

### Clinical target volume

The CTV defines the volume that includes the GTV and the surrounding tissues, accounting for the microscopic infiltration of the tumor at a certain probability level (CTV-P). The CTV‑N represents the regional lymph drainage area with an elevated risk for microscopic lymphatic metastasis [123]. The probability of subclinical tumor spread is based on histopathological evidence, insights gained from previous treatment outcomes, and consecutive failure pattern analysis of tumor recurrences. Unlike the GTV, the CTV cannot be directly identified using current imaging techniques, adding further complexity to the definition of the CTV. Unkelbach et al. provided a comprehensive overview of the role of computational methods in the automation of CTV delineation [101, 124].

Employing deep learning methods for automating target volume delineation process can assist in improving consistency and achieving a better balance between undertreating microscopic disease and unnecessary radiation exposure of normal tissues. Deep learning approaches have demonstrated efficacy in automating CTV delineation for various tumor types such as head and neck [125–127], esophageal [128], oropharyngeal [129], lung [130], rectal [2], breast [131–133], and cervical cancers [134–137].

**Fig. 5:**
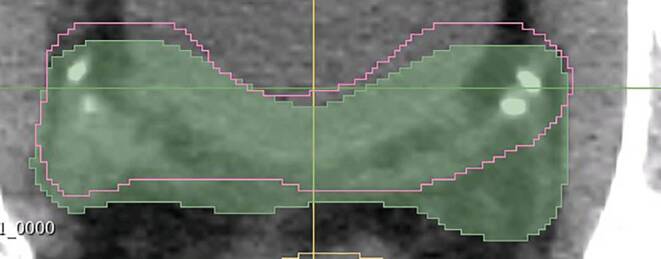
**Automatic segmentation for prostate salvage radiotherapy clinical target volume (CTV)**. Ground truth (*green area*) and automatic segmentation based on an in-house-developed U‑Net (*pink outline*) for prostate salvage radiotherapy

While the CTV‑P is often derived by geometric expansion of the GTV, consideration of anatomical barriers that impede tumor spread is crucial in CTV definition. Shusharina et al. demonstrated a combined approach for anatomically constrained 3D expansion based on Dijkstra’s shortest path search algorithm and autosegmentation of anatomical barriers with deep learning [138]. Aside from excluding anatomical structures from the CTV, autosegmentation becomes particularly valuable when incorporating references to anatomical structures such as entire organs and clearly defined lymph node regions in the delineation of CTVs. Aldoj et al. successfully implemented an algorithm inspired by the DenseNet and U‑Net architecture for autosegmentation of the prostate and prostate zones using MRI images, achieving a DSC of 0.921 for the whole gland [139]. Even CT-based segmentation for salvage prostate radiotherapy with 3D nnU-Net provides promising results (Fig. 5).

Deep learning-based autosegmentation also proved effective in the delineation of lymph node level target volumes. Employing an end-to-end deep deconvolutional neural network architecture, accurate segmentation of the CTV including the high-risk lymphatic drainage areas with a DSC of 0.826 was achieved for nasopharyngeal carcinoma using planning CT images [125]. Furthermore, application of deep learning-based autosegmentation extended to individual lymph node level target volumes in head and neck cancers using the U‑Net model and CT scans achieved DSC scores between 0.81 and 0.90; 99% of the autosegmented target volumes were deemed clinically acceptable or required only minor edits [127]. For cervical cancer, the development of a deep learning tool based on VB-Net enabled the delineation of CTVs within the pelvic lymphatic drainage area and parametrial region for definitive and postoperative radiotherapy with DSC scores ranging from 0.70 to 0.88, decreasing the mean contouring time by 9.8 min, which reflected a 25% reduction. The contouring accuracy was comparable to that of senior radiation oncologists, and deep learning assistance improved the performance of junior radiation oncologists [140]. For planning CTs in breast cancer, using a 3D-CNN for segmentation of the CTV including the lymphatic drainage, guideline consistency improved from 77.14% to 90.71% while 24 min were saved on average [133]. Deep learning-assisted contouring based on CT images for postoperative lung cancer improved contour accuracy as indicated by a higher DSC of 0.75 compared to 0.72 for manual segmentation and decreased inter-observer variability, showcasing a smaller coefficient of variation of 0.129 compared to 0.183 and standard distance deviation of 0.47 compared to 0.72. Moreover, a 35% time saving was observed [141]. In a multi-center study, automated detection and segmentation of lymph nodes in rectal cancer were achieved using multi parametric MRI and the Mask R‑CNN architecture, yielding a DSC of 0.81 to 0.82, requiring only 1.3 s per case compared to 200 s per case for radiologists[142].

### Challenges prior to clinical implementation

Despite the successes, several challenges still remain in the field of deep learning-based autosegmentation [143]. First, the generalization of these models across different patient populations and imaging modalities remains a major concern. The variability in imaging protocols, patient anatomy, and tumor characteristics pose a significant challenge to the robustness of deep learning models, making it difficult to adequately train autosegmentation models on larger population pools [144]. Second, the accuracy of segmenting structures with significant variability poses a challenge. This necessitates ongoing research to enhance model training with larger datasets and innovative approaches [18]. Additionally, the integration of these models into varied clinical settings highlights the need for adaptable and flexible systems that can cater to different institutional protocols and patient populations [145, 146]. However, due to privacy concerns and data-sharing regulations, gathering large-scale datasets with a wide range of patient demographics and sufficient representation of various cancer entities to develop such flexible models remains difficult.

Before integrating commercial systems into the clinical workflow, it has to be taken into account that the model might be trained on a dataset that varies considerably from the target patients [147]. Differences can include image quality and acquisition parameters as well as institutional protocols. At the patient level, demographic characteristics, the contouring style of the planning physician, and the choice of contouring guidelines contribute to the uncertainty. If the difference becomes significant, the segmentation quality can deteriorate, which can severely impact treatment quality. Therefore, every radiotherapy team should undergo thorough education about the use and especially the limits of autosegmentation to be aware of its shortcomings.

To navigate these obstacles successfully, Vandewinckele et al. proposed a two-step workflow for AI systems [87]:

In the first phase, i.e., the commission, a thorough test phase of the model should be performed with an in-house test set. This allows for a basic evaluation of the segmentation quality and, thus, its compatibility with the given data. The test set should be kept for later quality assessment (QA). In addition, this test puts the promoted metrics in perspective and unveils inter-observer variability within the team [148–150]. Only after reliable performance is observed should the model be integrated into the clinical workflow.

During the second phase, i.e., the implementation, all future users are to be instructed in detail about the possibilities and shortcomings of the model to be used. Segmentation for OARs is considered a suitable task to start with. Still, each segmentation needs to be reviewed and, if necessary, edited. For further model improvement, an ongoing logging of necessary modifications to the segmentation is advised [87]. Even after successful implementation, manual review is compulsory to ensure a case-specific quality assessment. During software updates or changes in the institution’s workflow (different imaging protocols or machinery), the quality or even the basic operations of the model can be impeded. To mitigate these risks, the model performance should be tested in regular check-ups based on the test set by the commission. Additionally, automatic model-integrated QA tools exist in commercial applications to guarantee a reliable and valid functioning of the model itself [151–153]. In predefined timeframes, these QA tools test the model for internal robustness and an at least level performance of segmentation. The results of these routine QA assessments should be logged for observation of long-term improvement and trends. Due to the aforementioned variables in the model training set and at the patient level, review by a physician can not be substituted [87].

## Outlook

### Target volume definition 2.0

Given the limitations of imaging methods in detecting the extent of microscopic tumor spread, mathematical modeling of tumor growth can play a crucial role in defining individual CTVs, as elaborated in the following section.

#### Tumor growth models

Over the past decade, mathematical tumor growth modeling has been proposed as an alternative method to guide radiotherapy dose distribution, especially in the case of brain tumors, due to their enclosed environment. Employing mathematical concepts, this approach aims to estimate tumor cell density over the whole brain, which cannot be obtained directly from conventional imaging. This additional information can be used to warp the CTV towards areas of higher tumor cell density, which would be neglected by common CTV design in the case of a location more distant from the tumor core or a benign appearance in conventional MRI.

**Fig. 6:**
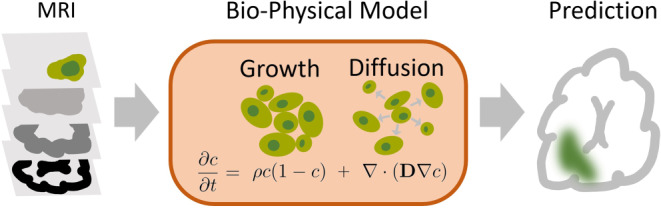
Fitting a biophysical growth model to tumor concentration estimation. Based on MRI scans, enhancing and edema regions are segmented (Sect. 3.2) and subsequently fitted to a biophysical model. The shown model (*orange box*) is governed by a partial differential equation describing the change in tumor cell concentration over time $\partial c/\partial t$. $\rho$ represents the proliferation rate and $D$ the diffusion coefficient of the tumor. The result of the simulation contains the estimated tumor concentration in each voxel

Tumor growth can be effectively modeled using partial differential equations. Thereby, the change in tumor concentration over time is described by various factors. For example, in the simple Fisher–Kolmogrov model (Fig. 6), a logistics growth term is combined with a diffusion term known as a reaction–diffusion model. Arbitrarily complex additions can be made, taking various biological factors into account including necrosis, tumor genetics, advection (tissue shifting), and surrounding tissue. A further difficulty lies in fitting these models to measured patient data. Therefore, methods ranging from classical Monte Carlo sampling to deep learning [154–156] are applied.

In a recent study, a novel deep learning-enhanced growth model was tested for its clinical applicability in radiotherapy planning. The model was trained on a dataset of numerical simulations, which eliminates the need for large datasets of longitudinal brain tumor images and is able to predict the individual spatial distribution of the tumor on MRI data from a single time point, namely preoperatively, and two commonly available sequences only (contrast-enhanced T1 and T2/FLAIR scans) [154]. In a clinical pilot study, alternative clinical target volumes based on the model’s estimated tumor cell density were tested for their superiority over conventional radiotherapy planning [157]. Depending on the chosen cutoff value of tumor cell density, a significant improvement in coverage of later tumor recurrence was observed without a significantly increased total radiation volume, thus setting a starting point for further clinical implementation. Other studies have made joint use of metabolic and structural imaging, i.e., [^18^F]Fluorethyltyrosine(FET)-PET and MRI, to calibrate the patient-specific tumor growth model that can be used for personalized radiotherapy design [155] or have combined growth modeling with an exponential cell survival model to describe the effect of radiotherapy [158]. Another promising approach for advanced radiotherapy delineation is the integration of diffusion tensor imaging into tumor growth modeling to account for anisotropic tumor spread along fiber tracts [159, 160].

##### Clinical implementation

Several problems exist that hinder the application of tumor growth modeling in clinical practice. Due to limited validation data, it is unclear to what degree deterministic models can predict tumor concentrations. Therefore, the adequate complexity of the models, which should contain enough biologically relevant information while not overfitting the data, has yet to be determined.

#### AI-based tumor detection for personalized target volume definition

##### Detection of lymph node metastasis

Detection of lymph node (LN) metastasis is an important part of the staging examinations preceding tumor treatment. The affection of LNs can lead to an adapted CTV to cover the metastases, an increase in applied dose as an integrated boost, or even alter the overall treatment choice. However, detecting metastases in these LNs is a particularly challenging task in daily clinical practice [161].

Promising results in terms of predicting LN status by the use of AI techniques such as *radiomics* (hard-coded quantitative imaging features that are fed into conventional machine learning models) have been achieved for different tumor histologies [162–164]. Several different approaches have been published in recent years, ranging from improving the LN classification from PET/CT to directly predicting LN status from conventional CT imaging alone and predicting PET/CT from conventional CT.

Rogasch et al. showed that a machine learning model based on routinely available LN features from [^18^F]Fluorodeoxyglucose(FDG)-PET/CT (such as size and SUVmax) improves the accuracy of mediastinal LN staging in lung cancer patients compared to established visual assessment criteria such as comparing LN tracer uptake to the mediastinum [165].

While they were unable to predict the primary tumor histology, Flechsig et al. demonstrated the ability of density-based CT histogram profiling to differentiate benign from malignant LNs in lung cancer patients [166]. They furthermore proposed a possible cutoff value of 20 Hounsfield units to differentiate LNs, especially in cases with equivocal tracer uptake in PET/CT.

After treatment of prostate cancer with radical prostatectomy, the pelvic LNs are a common site of recurrence. While prostate-specific membrane antigen (PSMA) PET/CT remains unsurpassed in its diagnostic capabilities, AI analysis of conventional CT imaging has been shown to be superior to conventional features (e.g., LN short diameter) for predicting recurrence in LNs and, therefore, may aid in CTV adaption [167]. Similar results have been achieved for cervical LNs in patients with oral squamous cell carcinoma [168].

Due to the high cost and limited availability of PSMA PET/CT scans, researchers have attempted to predict PSMA PET/CT positivity using CT imaging alone in prostate cancer patients [169]. In contrast to Peeken et al.’s method described above, this study predicted PET/CT positivity instead of histological grading.

##### Tumor infiltration

In cases where the exact extent of a tumor cannot be determined completely by consulting the currently available imaging modalities, neural networks and radiomics can be used to predict areas with a high likelihood of recurrence [170]. Especially for glioblastoma, where the infiltration of the tumor into the surrounding edema remains uncertain, multiparametric pattern analysis shows promising results to assess the spatial extent of the tumor [171]. Other methods include deep learning algorithms to process MR sequences to generate a more detailed representation of tumor infiltration, as shown for diffusion tensor imaging in glioblastoma patients [170]. These methods might not only enable radiation oncologists to target possible sites of recurrence more securely but, above all, they would allow for more personalized treatment with dose escalation in volumes that models deem at risk of recurrence and, at the same time, dose deescalation in regions that are predicted to have a minor risk of infiltration.

### One-stop-shop segmentation and treatment planning

Considering the performance of contemporary algorithms and the speed of the development of AI algorithms, one can envision a near future in which the complete segmentation process can be performed in a fully automated fashion for standardized treatment scenarios. Later on, autocontouring foundation models paired with large language models may open up the possibility of tailoring volumes to specific accompanying clinical factors and any individual clinical situation of specific patients.

As discussed in another review in this issue, AI can also take over the treatment planning process. As a consequence, it is only a matter of time before a future AI model covers the full process from segmentation to radiotherapy plan generation in an one-stop-shop approach. For instance, Xiang et al. developed an AI-based concept to accomplish the full process of radiotherapy planning for rectal cancer on CT scans. For all patients, they reached a minimum PTV DSC of 0.85 and mean OAR DSC of 0.75. After just one mouse click, plans were ready in 7  min, followed by expert contour modifications with an average duration of 13.3 min and reoptimization with 5 min. However, modifications were only necessary in 20% of the plans [172]. When using high-quality cone-beam CT or MRI from a linear accelerator device as the basis for treatment planning, it will soon be feasible to perform the whole treatment planning and treatment delivery process in a one-stop-shop treatment session, thereby enabling same-day treatments.

## Conclusion

In summary, the swift evolution of AI has propelled deep learning autosegmentation into the forefront of radiotherapy treatment planning. Its demonstrated potential to significantly reshape the landscape of the radiation oncology workflow is underscored by the initial deployment of models in clinical practice, particularly for OARs. The advent of models approaching or on the cusp of clinical use for GTV and CTV segmentation marks a pivotal step toward comprehensive integration.

This review highlights discrete benefits arising from the adoption of deep learning autosegmentation, emphasizing its role in enhancing segmentation efficiency, thus promoting consistency and mitigating inter-operator variability. As evidenced by its successful application in OAR segmentation and promising strides in GTV and CTV modeling, AI stands as a transformative force in achieving more precise and reproducible radiotherapy plans.

Looking ahead, the continuous technical advancements in deep learning are poised to unlock even broader applications. This encompasses not only improvements in segmentation performance but also the expansion of the number of addressed entities. Moreover, the integration of superior tumor detection methods represents a key frontier for further refinement.

In conclusion, the intersection of deep learning autosegmentation and radiotherapy holds immense potential for advancing the field to foster efficiency and ultimately improve patient outcomes. Though more and more processes might become automatized, every automated segmentation still has to undergo critical evaluation by an approved radiation oncology expert.

## Declarations

### Editorial Policies

Springer journals and proceedings: https://www.springer.com/gp/editorial-policies

## References

[1] G. Samarasinghe, M. Jameson, S. Vinod, M. Field, J. Dowling, A. Sowmya, and L. Holloway, “Deep learning for segmentation in radiation therapy planning: a review,” *Journal of Medical Imaging and Radiation Oncology*, vol. 65, no. 5, pp. 578–595, 2021. 10.1111/1754-9485.13286 34313006

[2] K. Men, J. Dai, and Y. Li, “Automatic segmentation of the clinical target volume and organs at risk in the planning CT for rectal cancer using deep dilated convolutional neural networks,” *Medical physics*, vol. 44, no. 12, pp. 6377–6389, 2017. 10.1002/mp.12602 28963779

[3] K. Harrison, H. Pullen, C. Welsh, O. Oktay, J. Alvarez-Valle, and R. Jena, “Machine Learning for Auto-Segmentation in Radiotherapy Planning,” *Clinical Oncology (Royal College of Radiologists (Great Britain))*, vol. 34, no. 2, pp. 74–88, 2022. 10.1016/j.clon.2021.12.003 34996682

[4] J. Wong, V. Huang, J. A. Giambattista, T. Teke, C. Kolbeck, J. Giambattista, and S. Atrchian, “Training and Validation of Deep Learning-Based Auto-Segmentation Models for Lung Stereotactic Ablative Radiotherapy Using Retrospective Radiotherapy Planning Contours,” *Frontiers in Oncology*, vol. 11, p. 626499, 2021. 10.3389/fonc.2021.626499 PMC8215371 34164335

[5] B. Ibragimov and L. Xing, “Segmentation of organs-at-risks in head and neck CT images using convolutional neural networks,” *Medical Physics*, vol. 44, no. 2, pp. 547–557, 2017. 10.1002/mp.12045 PMC5383420 28205307

[6] J. Wong, V. Huang, D. Wells, J. Giambattista, J. Giambattista, C. Kolbeck, K. Otto, E. P. Saibishkumar, and A. Alexander, “Implementation of deep learning-based auto-segmentation for radiotherapy planning structures: a workflow study at two cancer centers,” *Radiation Oncology*, vol. 16, no. 1, p. 101, 2021. 10.1186/s13014-021-01831-4 PMC8186196 34103062

[7] J. C. Korte, N. Hardcastle, S. P. Ng, B. Clark, T. Kron, and P. Jackson, “Cascaded deep learning-based auto-segmentation for head and neck cancer patients: Organs at risk on T2-weighted magnetic resonance imaging,” *Medical Physics*, vol. 48, no. 12, pp. 7757–7772, 2021. https://onlinelibrary.wiley.com/doi/pdf/10.1002/mp.15290. 10.1002/mp.15290 34676555

[8] K. Harrison, H. Pullen, C. Welsh, O. Oktay, J. Alvarez-Valle, and R. Jena, “Machine learning for auto-segmentation in radiotherapy planning,” *Clinical Oncology*, vol. 34, no. 2, pp. 74–88, 2022. 10.1016/j.clon.2021.12.003 34996682

[9] K. Elagouni, C. Ciofolo-Veit, and B. Mory, “Automatic segmentation of pathological tissues in cardiac MRI,” in *2010 IEEE International Symposium on Biomedical Imaging: From Nano to Macro*, pp. 472–475, 2010. ISSN: 1945-8452.

[10] O. Alirr and A. A. Abd. Rahni, “Automatic liver segmentation from ct scans using intensity analysis and level-set active contours,” *Journal of Engineering Science and Technology*, vol. 13, pp. 3821–3839, 2018.

[11] N. Sarkalkan, H. Weinans, and A. A. Zadpoor, “Statistical shape and appearance models of bones,” *Bone*, vol. 60, pp. 129–140, 2014. 10.1016/j.bone.2013.12.006 24334169

[12] C. M. Engstrom, J. Fripp, V. Jurcak, D. G. Walker, O. Salvado, and S. Crozier, “Segmentation of the quadratus lumborum muscle using statistical shape modeling,” *Journal of Magnetic Resonance Imaging*, vol. 33, no. 6, pp. 1422–1429, 2011. https://onlinelibrary.wiley.com/doi/pdf/10.1002/jmri.22188. 10.1002/jmri.22188 21591012

[13] K. D. Fritscher, M. Peroni, P. Zaffino, M. F. Spadea, R. Schubert, and G. Sharp, “Automatic segmentation of head and neck CT images for radiotherapy treatment planning using multiple atlases, statistical appearance models, and geodesic active contours,” *Medical Physics*, vol. 41, no. 5, p. 051910, 2014. 10.1118/1.4871623 PMC4000401 24784389

[14] C. L. Brouwer, R. J. H. M. Steenbakkers, J. Bourhis, W. Budach, C. Grau, V. Grégoire, M. van Herk, A. Lee, P. Maingon, C. Nutting, B. O’Sullivan, S. V. Porceddu, D. I. Rosenthal, N. M. Sijtsema, and J. A. Langendijk, “CT-based delineation of organs at risk in the head and neck region: DAHANCA, EORTC, GORTEC, HKNPCSG, NCIC CTG, NCRI, NRG Oncology and TROG consensus guidelines,” *Radiotherapy and Oncology: Journal of the European Society for Therapeutic Radiology and Oncology*, vol. 117, no. 1, pp. 83–90, 2015. 10.1016/j.radonc.2015.07.041 26277855

[15] T. Vrtovec, D. Močnik, P. Strojan, F. Pernuš, and B. Ibragimov, “Auto-segmentation of organs at risk for head and neck radiotherapy planning: from atlas-based to deep learning methods,” *Medical physics*, vol. 47, no. 9, pp. e929–e950, 2020. 10.1002/mp.14320 32510603

[16] X. Wu, J. K. Udupa, Y. Tong, D. Odhner, G. V. Pednekar, C. B. Simone, D. McLaughlin, C. Apinorasethkul, O. Apinorasethkul, J. Lukens, D. Mihailidis, G. Shammo, P. James, A. Tiwari, L. Wojtowicz, J. Camaratta, and D. A. Torigian, “AAR-RT – A system for auto-contouring organs at risk on CT images for radiation therapy planning: Principles, design, and large-scale evaluation on head-and-neck and thoracic cancer cases,” *Medical Image Analysis*, vol. 54, pp. 45–62, 2019. 10.1016/j.media.2019.01.008 PMC6499546 30831357

[17] B. Schipaanboord, D. Boukerroui, D. Peressutti, J. van Soest, T. Lustberg, T. Kadir, A. Dekker, W. van Elmpt, and M. Gooding, “Can Atlas-Based Auto-Segmentation Ever Be Perfect? Insights From Extreme Value Theory,” *IEEE transactions on medical imaging*, vol. 38, no. 1, pp. 99–106, 2019. 10.1109/TMI.2018.2856464 30010554

[18] P. Meyer, V. Noblet, C. Mazzara, and A. Lallement, “Survey on deep learning for radiotherapy,” *Computers in Biology and Medicine*, vol. 98, pp. 126–146, 2018. 10.1016/j.compbiomed.2018.05.018 29787940

[19] O. Ronneberger, P. Fischer, and T. Brox, “U-net: Convolutional networks for biomedical image segmentation,” in *Medical Image Computing and Computer-Assisted Intervention–MICCAI 2015: 18th International Conference, Munich, Germany, October 5‑9, 2015, Proceedings, Part III 18*, pp. 234–241, Springer, 2015.

[20] K. He, X. Zhang, S. Ren, and J. Sun, “Deep residual learning for image recognition,” in *Proceedings of the IEEE conference on computer vision and pattern recognition*, pp. 770–778, 2016.

[21] Z. Huang, H. Wang, J. Ye, J. Niu, C. Tu, Y. Yang, S. Du, Z. Deng, L. Gu, and J. He, “Revisiting nnu-net for iterative pseudo labeling and efficient sliding window inference,” in *MICCAI Challenge on Fast and Low-Resource Semi-supervised Abdominal Organ Segmentation*, pp. 178–189, Springer, 2022.

[22] H. M. Luu and S.-H. Park, “Extending nn-UNet for brain tumor segmentation,” in *International MICCAI Brainlesion Workshop*, pp. 173–186, Springer, 2021.

[23] C. González, A. Ranem, D. Pinto dos Santos, A. Othman, and A. Mukhopadhyay, “Lifelong nnu-net: a framework for standardized medical continual learning,” *Scientific Reports*, vol. 13, no. 1, p. 9381, 2023. 10.1038/s41598-023-34484-2 PMC10256748 37296233

[24] A. Dosovitskiy, L. Beyer, A. Kolesnikov, D. Weissenborn, X. Zhai, T. Unterthiner, M. Dehghani, M. Minderer, G. Heigold, S. Gelly, et al., “An image is worth 16x16 words: Transformers for image recognition at scale,” *arXiv preprint arXiv:2010.11929*, 2020.

[25] A. Vaswani, N. Shazeer, N. Parmar, J. Uszkoreit, L. Jones, A. N. Gomez, Ł. Kaiser, and I. Polosukhin, “Attention is all you need,” *Advances in neural information processing systems*, vol. 30, 2017.

[26] S. Khan, M. Naseer, M. Hayat, S. W. Zamir, F. S. Khan, and M. Shah, “Transformers in vision: A survey,” *ACM computing surveys (CSUR)*, vol. 54, no. 10s, pp. 1–41, 2022.

[27] Y. Tang, D. Yang, W. Li, H. R. Roth, B. Landman, D. Xu, V. Nath, and A. Hatamizadeh, “Self-supervised pre-training of swin transformers for 3d medical image analysis,” in *Proceedings of the IEEE/CVF Conference on Computer Vision and Pattern Recognition*, pp. 20730–20740, 2022.

[28] A. Hatamizadeh, Y. Tang, V. Nath, D. Yang, A. Myronenko, B. Landman, H. R. Roth, and D. Xu, “Unetr: Transformers for 3d medical image segmentation,” in *Proceedings of the IEEE/CVF winter conference on applications of computer vision*, pp. 574–584, 2022.

[29] H.-Y. Zhou, J. Guo, Y. Zhang, L. Yu, L. Wang, and Y. Yu, “nnformer: Interleaved transformer for volumetric segmentation,” *arXiv preprint arXiv:2109.03201*, 2021.

[30] Z. Zhou, V. Sodha, J. Pang, M. B. Gotway, and J. Liang, “Models genesis,” *Medical image analysis*, vol. 67, p. 101840, 2021. 10.1016/j.media.2020.101840 PMC7726094 33188996

[31] J. Liu, Y. Zhang, J.-N. Chen, J. Xiao, Y. Lu, B. A Landman, Y. Yuan, A. Yuille, Y. Tang, and Z. Zhou, “Clip-driven universal model for organ segmentation and tumor detection,” in *Proceedings of the IEEE/CVF International Conference on Computer Vision*, pp. 21152–21164, 2023. 10.1109/iccv51070.2023.01934 PMC13332747 42403563

[32] A. Radford, J. W. Kim, C. Hallacy, A. Ramesh, G. Goh, S. Agarwal, G. Sastry, A. Askell, P. Mishkin, J. Clark, et al., “Learning transferable visual models from natural language supervision,” in *International conference on machine learning*, pp. 8748–8763, PMLR, 2021.

[33] V. I. Butoi, J. J. G. Ortiz, T. Ma, M. R. Sabuncu, J. Guttag, and A. V. Dalca, “Universeg: Universal medical image segmentation,” *arXiv preprint arXiv:2304.06131*, 2023.

[34] L. Liu, J. M. Wolterink, C. Brune, and R. N. Veldhuis, “Anatomy-aided deep learning for medical image segmentation: a review,” *Physics in Medicine & Biology*, vol. 66, no. 11, p. 11TR01, 2021. 10.1088/1361-6560/abfbf4 33906186

[35] N. Homayounfar, Y. Xiong, J. Liang, W.-C. Ma, and R. Urtasun, “Levelset r‑cnn: A deep variational method for instance segmentation,” in *Computer Vision–ECCV 2020: 16th European Conference, Glasgow, UK, August 23–28, 2020, Proceedings, Part XXIII 16*, pp. 555–571, Springer, 2020.

[36] J. Ding, Y. Zhang, A. Amjad, J. Xu, D. Thill, and X. A. Li, “Automatic contour refinement for deep learning auto-segmentation of complex organs in mri-guided adaptive radiation therapy,” *Advances in Radiation Oncology*, vol. 7, no. 5, p. 100968, 2022. 10.1016/j.adro.2022.100968 PMC9280040 35847549

[37] B. Zhang, Y. Wang, C. Ding, Z. Deng, L. Li, Z. Qin, Z. Ding, L. Bian, and C. Yang, “Multi-scale feature pyramid fusion network for medical image segmentation,” *International Journal of Computer Assisted Radiology and Surgery*, vol. 18, no. 2, pp. 353–365, 2023. 10.1007/s11548-022-02738-5 36042149

[38] L. P. Kaelbling, M. L. Littman, and A. W. Moore, “Reinforcement learning: A survey,” *Journal of artificial intelligence research*, vol. 4, pp. 237–285, 1996.

[39] X. Liao, W. Li, Q. Xu, X. Wang, B. Jin, X. Zhang, Y. Wang, and Y. Zhang, “Iteratively-refined interactive 3d medical image segmentation with multi-agent reinforcement learning,” in *Proceedings of the IEEE/CVF conference on computer vision and pattern recognition*, pp. 9394–9402, 2020.

[40] C. Ma, Q. Xu, X. Wang, B. Jin, X. Zhang, Y. Wang, and Y. Zhang, “Boundary-aware supervoxel-level iteratively refined interactive 3d image segmentation with multi-agent reinforcement learning,” *IEEE Transactions on Medical Imaging*, vol. 40, no. 10, pp. 2563–2574, 2020. 10.1109/TMI.2020.3048477 33382649

[41] R. Tomsett, D. Braines, D. Harborne, A. Preece, and S. Chakraborty, “Interpretable to whom? a role-based model for analyzing interpretable machine learning systems,” *arXiv preprint arXiv:1806.07552*, 2018.

[42] L. H. Gilpin, D. Bau, B. Z. Yuan, A. Bajwa, M. Specter, and L. Kagal, “Explaining explanations: An overview of interpretability of machine learning,” in *2018 IEEE 5th International Conference on data science and advanced analytics (DSAA)*, pp. 80–89, IEEE, 2018.

[43] M. Nauta, J. Trienes, S. Pathak, E. Nguyen, M. Peters, Y. Schmitt, J. Schlötterer, M. van Keulen, and C. Seifert, “From anecdotal evidence to quantitative evaluation methods: A systematic review on evaluating explainable ai,” *ACM Computing Surveys*, vol. 55, no. 13s, pp. 1–42, 2023.

[44] K. Borys, Y. A. Schmitt, M. Nauta, C. Seifert, N. Krämer, C. M. Friedrich, and F. Nensa, “Explainable ai in medical imaging: An overview for clinical practitioners–saliency-based xai approaches,” *European journal of radiology*, p. 110787, 2023. 10.1016/j.ejrad.2023.110787 37001254

[45] M. D. Zeiler and R. Fergus, “Visualizing and understanding convolutional networks,” in *Computer Vision–ECCV 2014: 13th European Conference, Zurich, Switzerland, September 6‑12, 2014, Proceedings, Part I 13*, pp. 818–833, Springer, 2014.

[46] M. T. Ribeiro, S. Singh, and C. Guestrin, “why should i trust you?” explaining the predictions of any classifier,” in *Proceedings of the 22nd ACM SIGKDD international conference on knowledge discovery and data mining*, pp. 1135–1144, 2016.

[47] S. M. Lundberg and S.-I. Lee, “A unified approach to interpreting model predictions,” *Advances in neural information processing systems*, vol. 30, 2017.

[48] V. Petsiuk, A. Das, and K. Saenko, “Rise: Randomized input sampling for explanation of black-box models,” *arXiv preprint arXiv:1806.07421*, 2018.

[49] A. Das and P. Rad, “Opportunities and challenges in explainable artificial intelligence (xai): A survey,” *arXiv preprint arXiv:2006.11371*, 2020.

[50] M. Sundararajan, A. Taly, and Q. Yan, “Axiomatic attribution for deep networks,” in *International conference on machine learning*, pp. 3319–3328, PMLR, 2017.

[51] J. T. Springenberg, A. Dosovitskiy, T. Brox, and M. Riedmiller, “Striving for simplicity: The all convolutional net,” *arXiv preprint arXiv:1412.6806*, 2014.

[52] R. R. Selvaraju, M. Cogswell, A. Das, R. Vedantam, D. Parikh, and D. Batra, “Grad-cam: Visual explanations from deep networks via gradient-based localization,” in *Proceedings of the IEEE international conference on computer vision*, pp. 618–626, 2017.

[53] R. A. Zeineldin, M. E. Karar, Z. Elshaer, ·. J. Coburger, C. R. Wirtz, O. Burgert, and F. Mathis-Ullrich, “Explainability of deep neural networks for mri analysis of brain tumors,” *International journal of computer assisted radiology and surgery*, vol. 17, no. 9, pp. 1673–1683, 2022. 10.1007/s11548-022-02619-x PMC9463287 35460019

[54] K. Borys, Y. A. Schmitt, M. Nauta, C. Seifert, N. Krämer, C. M. Friedrich, and F. Nensa, “Explainable ai in medical imaging: An overview for clinical practitioners–beyond saliency-based xai approaches,” *European journal of radiology*, p. 110786, 2023. 10.1016/j.ejrad.2023.110786 36990051

[55] M. Pocevičiūtė, G. Eilertsen, and C. Lundström, “Survey of XAI in digital pathology,” *Artificial intelligence and machine learning for digital pathology: state-of-the-art and future challenges*, pp. 56–88, 2020.

[56] A. Der Kiureghian and O. Ditlevsen, “Aleatory or epistemic? does it matter?,” *Structural safety*, vol. 31, no. 2, pp. 105–112, 2009.

[57] Y. Kwon, J.-H. Won, B. J. Kim, and M. C. Paik, “Uncertainty quantification using bayesian neural networks in classification: Application to biomedical image segmentation,” *Computational Statistics & Data Analysis*, vol. 142, p. 106816, 2020.

[58] S. Gao, H. Zhou, Y. Gao, and X. Zhuang, “Bayeseg: Bayesian modeling for medical image segmentation with interpretable generalizability,” *arXiv preprint arXiv:2303.01710*, 2023. 10.1016/j.media.2023.102889 37467643

[59] B. Lakshminarayanan, A. Pritzel, and C. Blundell, “Simple and scalable predictive uncertainty estimation using deep ensembles,” *Advances in neural information processing systems*, vol. 30, 2017.

[60] Y. Gal and Z. Ghahramani, “Dropout as a bayesian approximation: Representing model uncertainty in deep learning,” in *international conference on machine learning*, pp. 1050–1059, PMLR, 2016.

[61] S. Yang and T. Fevens, “Uncertainty quantification and estimation in medical image classification,” in *Artificial Neural Networks and Machine Learning–ICANN 2021: 30th International Conference on Artificial Neural Networks, Bratislava, Slovakia, September 14–17, 2021, Proceedings, Part III 30*, pp. 671–683, Springer, 2021.

[62] Y. Ovadia, E. Fertig, J. Ren, Z. Nado, D. Sculley, S. Nowozin, J. Dillon, B. Lakshminarayanan, and J. Snoek, “Can you trust your model’s uncertainty? Evaluating predictive uncertainty under dataset shift,” *Advances in neural information processing systems*, vol. 32, 2019.

[63] G. Wang, W. Li, M. Aertsen, J. Deprest, S. Ourselin, and T. Vercauteren, “Aleatoric uncertainty estimation with test-time augmentation for medical image segmentation with convolutional neural networks,” *Neurocomputing*, vol. 338, pp. 34–45, 2019. 10.1016/j.neucom.2019.01.103 PMC6783308 31595105

[64] A. Kendall and Y. Gal, “What uncertainties do we need in bayesian deep learning for computer vision?,” *Advances in neural information processing systems*, vol. 30, 2017.

[65] C. Guo, G. Pleiss, Y. Sun, and K. Q. Weinberger, “On calibration of modern neural networks,” in *International conference on machine learning*, pp. 1321–1330, PMLR, 2017.

[66] I. Goodfellow, J. Pouget-Abadie, M. Mirza, B. Xu, D. Warde-Farley, S. Ozair, A. Courville, and Y. Bengio, “Generative adversarial nets,” *Advances in neural information processing systems*, vol. 27, 2014.

[67] J. Sohl-Dickstein, E. Weiss, N. Maheswaranathan, and S. Ganguli, “Deep unsupervised learning using nonequilibrium thermodynamics,” in *International conference on machine learning*, pp. 2256–2265, PMLR, 2015.

[68] J. Ho, A. Jain, and P. Abbeel, “Denoising diffusion probabilistic models,” *Advances in neural information processing systems*, vol. 33, pp. 6840–6851, 2020.

[69] J. Song, C. Meng, and S. Ermon, “Denoising diffusion implicit models,” in *International Conference on Learning Representations*, 2020.

[70] Y. Zhang, H. Ling, J. Gao, K. Yin, J.-F. Lafleche, A. Barriuso, A. Torralba, and S. Fidler, “Datasetgan: Efficient labeled data factory with minimal human effort,” in *Proceedings of the IEEE/CVF Conference on Computer Vision and Pattern Recognition*, pp. 10145–10155, 2021.

[71] D. Li, J. Yang, K. Kreis, A. Torralba, and S. Fidler, “Semantic segmentation with generative models: Semi-supervised learning and strong out-of-domain generalization,” in *Proceedings of the IEEE/CVF Conference on Computer Vision and Pattern Recognition*, pp. 8300–8311, 2021.

[72] R. Abdal, P. Zhu, N. J. Mitra, and P. Wonka, “Labels4free: Unsupervised segmentation using stylegan,” in *Proceedings of the IEEE/CVF International Conference on Computer Vision*, pp. 13970–13979, 2021.

[73] T. Karras, S. Laine, and T. Aila, “A style-based generator architecture for generative adversarial networks,” in *Proceedings of the IEEE/CVF conference on computer vision and pattern recognition*, pp. 4401–4410, 2019. 10.1109/TPAMI.2020.2970919 32012000

[74] J. Xu, S. Liu, A. Vahdat, W. Byeon, X. Wang, and S. De Mello, “Open-vocabulary panoptic segmentation with text-to-image diffusion models,” in *Proceedings of the IEEE/CVF Conference on Computer Vision and Pattern Recognition*, pp. 2955–2966, 2023.

[75] M. Rosnati, F. D. S. Ribeiro, M. Monteiro, D. C. de Castro, and B. Glocker, “Analysing the effectiveness of a generative model for semi-supervised medical image segmentation,” in *Machine Learning for Health*, pp. 290–310, PMLR, 2022.

[76] P. Moeskops, M. Veta, M. W. Lafarge, K. A. Eppenhof, and J. P. Pluim, “Adversarial training and dilated convolutions for brain mri segmentation,” in *Deep Learning in Medical Image Analysis and Multimodal Learning for Clinical Decision Support: Third International Workshop, DLMIA 2017, and 7th International Workshop, ML-CDS 2017, Held in Conjunction with MICCAI 2017, Québec City, QC, Canada, September 14, Proceedings 3*, pp. 56–64, Springer, 2017.

[77] Z. Li, Y. Wang, and J. Yu, “Brain tumor segmentation using an adversarial network,” in *Brainlesion: Glioma, Multiple Sclerosis, Stroke and Traumatic Brain Injuries: Third International Workshop, BrainLes 2017, Held in Conjunction with MICCAI 2017, Quebec City, QC, Canada, September 14, 2017, Revised Selected Papers 3*, pp. 123–132, Springer, 2018.

[78] M. Rezaei, K. Harmuth, W. Gierke, T. Kellermeier, M. Fischer, H. Yang, and C. Meinel, “A conditional adversarial network for semantic segmentation of brain tumor,” in *Brainlesion: Glioma, Multiple Sclerosis, Stroke and Traumatic Brain Injuries: Third International Workshop, BrainLes 2017, Held in Conjunction with MICCAI 2017, Quebec City, QC, Canada, September 14, 2017, Revised Selected Papers 3*, pp. 241–252, Springer, 2018.

[79] Y. Xue, T. Xu, H. Zhang, L. R. Long, and X. Huang, “Segan: Adversarial network with multi-scale l 1 loss for medical image segmentation,” *Neuroinformatics*, vol. 16, pp. 383–392, 2018. 10.1007/s12021-018-9377-x PMC13344194 29725916

[80] T. Eelbode, J. Bertels, M. Berman, D. Vandermeulen, F. Maes, R. Bisschops, and M. B. Blaschko, “Optimization for medical image segmentation: theory and practice when evaluating with dice score or jaccard index,” *IEEE Transactions on Medical Imaging*, vol. 39, no. 11, pp. 3679–3690, 2020. 10.1109/TMI.2020.3002417 32746113

[81] S. Thörnqvist, J. B. Petersen, M. Høyer, L. N. Bentzen, and L. P. Muren, “Propagation of target and organ at risk contours in radiotherapy of prostate cancer using deformable image registration,” *Acta Oncologica*, vol. 49, no. 7, pp. 1023–1032, 2010. 10.3109/0284186X.2010.503662 20831491

[82] D. P. Huttenlocher, G. A. Klanderman, and W. J. Rucklidge, “Comparing images using the hausdorff distance,” *IEEE Transactions on pattern analysis and machine intelligence*, vol. 15, no. 9, pp. 850–863, 1993.

[83] S. Nikolov, S. Blackwell, A. Zverovitch, R. Mendes, M. Livne, J. De Fauw, Y. Patel, C. Meyer, H. Askham, B. Romera-Paredes, et al., “Deep learning to achieve clinically applicable segmentation of head and neck anatomy for radiotherapy,” *arXiv preprint arXiv:1809.04430*, 2018. 10.2196/26151 PMC8314151 34255661

[84] J. Schlemper, O. Oktay, M. Schaap, M. Heinrich, B. Kainz, B. Glocker, and D. Rueckert, “Attention gated networks: Learning to leverage salient regions in medical images,” *Medical image analysis*, vol. 53, pp. 197–207, 2019. 10.1016/j.media.2019.01.012 PMC7610718 30802813

[85] W. Chen, Y. Li, B. A. Dyer, X. Feng, S. Rao, S. H. Benedict, Q. Chen, and Y. Rong, “Deep learning vs. atlas-based models for fast auto-segmentation of the masticatory muscles on head and neck ct images,” *Radiation Oncology*, vol. 15, no. 1, pp. 1–10, 2020. 10.1186/s13014-020-01617-0 PMC7372849 32690103

[86] W. J. Zabel, J. L. Conway, A. Gladwish, J. Skliarenko, G. Didiodato, L. Goorts-Matthews, A. Michalak, S. Reistetter, J. King, K. Nakonechny, et al., “Clinical evaluation of deep learning and atlas-based auto-contouring of bladder and rectum for prostate radiation therapy,” *Practical Radiation Oncology*, vol. 11, no. 1, pp. e80–e89, 2021. 10.1016/j.prro.2020.05.013 32599279

[87] L. Vandewinckele, M. Claessens, A. Dinkla, C. Brouwer, W. Crijns, D. Verellen, and W. van Elmpt, “Overview of artificial intelligence-based applications in radiotherapy: Recommendations for implementation and quality assurance,” *Radiotherapy and Oncology*, vol. 153, pp. 55–66, 2020. 10.1016/j.radonc.2020.09.008 32920005

[88] J. van der Veen, A. Gulyban, and S. Nuyts, “Interobserver variability in delineation of target volumes in head and neck cancer,” *Radiotherapy and Oncology*, vol. 137, pp. 9–15, 2019. 10.1016/j.radonc.2019.04.006 31048235

[89] X. A. Li, A. Tai, D. W. Arthur, T. A. Buchholz, S. Macdonald, L. B. Marks, J. M. Moran, L. J. Pierce, R. Rabinovitch, A. Taghian, et al., “Variability of target and normal structure delineation for breast cancer radiotherapy: an rtog multi-institutional and multiobserver study,” *International Journal of Radiation Oncology* Biology* Physics*, vol. 73, no. 3, pp. 944–951, 2009. 10.1016/j.ijrobp.2008.10.034 PMC2911777 19215827

[90] B. E. Nelms, W. A. Tomé, G. Robinson, and J. Wheeler, “Variations in the contouring of organs at risk: test case from a patient with oropharyngeal cancer,” *International Journal of Radiation Oncology* Biology* Physics*, vol. 82, no. 1, pp. 368–378, 2012. 10.1016/j.ijrobp.2010.10.019 21123004

[91] F. Kofler, J. Wahle, I. Ezhov, S. J. Wagner, R. Al-Maskari, E. Gryska, M. Todorov, C. Bukas, F. Meissen, T. Peng, et al., “Approaching peak ground truth,” in *2023 IEEE 20th International Symposium on Biomedical Imaging (ISBI)*, pp. 1–6, IEEE, 2023.

[92] M. V. Sherer, D. Lin, S. Elguindi, S. Duke, L.-T. Tan, J. Cacicedo, M. Dahele, and E. F. Gillespie, “Metrics to evaluate the performance of auto-segmentation for radiation treatment planning: A critical review,” *Radiotherapy and Oncology*, vol. 160, pp. 185–191, 2021. 10.1016/j.radonc.2021.05.003 PMC9444281 33984348

[93] J. Duan, M. E. Bernard, J. R. Castle, X. Feng, C. Wang, M. C. Kenamond, and Q. Chen, “Contouring quality assurance methodology based on multiple geometric features against deep learning auto-segmentation,” *Medical Physics*, 2023. 10.1002/mp.16299 PMC10175153 36788735

[94] F. Kofler, I. Ezhov, F. Isensee, F. Balsiger, C. Berger, M. Koerner, B. Demiray, J. Rackerseder, J. Paetzold, H. Li, et al., “Are we using appropriate segmentation metrics? Identifying correlates of human expert perception for CNN training beyond rolling the DICE coefficient,” *arXiv preprint arXiv:2103.06205*, 2021.

[95] Y. Fu, T. R. Mazur, X. Wu, S. Liu, X. Chang, Y. Lu, H. H. Li, H. Kim, M. C. Roach, L. Henke, et al., “A novel mri segmentation method using cnn-based correction network for mri-guided adaptive radiotherapy,” *Medical physics*, vol. 45, no. 11, pp. 5129–5137, 2018. 10.1002/mp.13221 30269345

[96] J. van der Veen, A. Gulyban, S. Willems, F. Maes, and S. Nuyts, “Interobserver variability in organ at risk delineation in head and neck cancer,” *Radiation Oncology*, vol. 16, pp. 1–11, 2021. 10.1186/s13014-020-01677-2 PMC8240214 34183040

[97] C. E. Cardenas, J. Yang, B. M. Anderson, L. E. Court, and K. B. Brock, “Advances in auto-segmentation,” in *Seminars in radiation oncology*, vol. 29, pp. 185–197, Elsevier, 2019. 10.1016/j.semradonc.2019.02.001 31027636

[98] H. Sartor, D. Minarik, O. Enqvist, J. Ulén, A. Wittrup, M. Bjurberg, and E. Trägårdh, “Auto-segmentations by convolutional neural network in cervical and anorectal cancer with clinical structure sets as the ground truth,” *Clinical and Translational Radiation Oncology*, vol. 25, pp. 37–45, 2020. 10.1016/j.ctro.2020.09.004 PMC7519211 33005756

[99] C. L. Brouwer, R. J. Steenbakkers, E. van den Heuvel, J. C. Duppen, A. Navran, H. P. Bijl, O. Chouvalova, F. R. Burlage, H. Meertens, J. A. Langendijk, et al., “3d variation in delineation of head and neck organs at risk,” *Radiation Oncology*, vol. 7, no. 1, pp. 1–10, 2012. 10.1186/1748-717X-7-32 PMC3337234 22414264

[100] J. Yang, B. M. Beadle, A. S. Garden, D. L. Schwartz, and M. Aristophanous, “ A multimodality segmentation framework for automatic target delineation in head and neck radiotherapy,” *Medical physics*, vol. 42, no. 9, pp. 5310–5320, 2015. 10.1118/1.4928485 PMC4545076 26328980

[101] J. Unkelbach, T. Bortfeld, C. E. Cardenas, V. Gregoire, W. Hager, B. Heijmen, R. Jeraj, S. S. Korreman, R. Ludwig, B. Pouymayou, et al., “The role of computational methods for automating and improving clinical target volume definition,” *Radiotherapy and Oncology*, vol. 153, pp. 15–25, 2020. 10.1016/j.radonc.2020.10.002 33039428

[102] V. M. Anaya, “A Geometric and Dosimetric Analysis of Limbus AI and AI-Rad Companion for Treatment Planning of H&N Cancer,”

[103] X. Feng, K. Qing, N. J. Tustison, C. H. Meyer, and Q. Chen, “Deep convolutional neural network for segmentation of thoracic organs-at-risk using cropped 3d images,” *Medical physics*, vol. 46, no. 5, pp. 2169–2180, 2019. 10.1002/mp.13466 30830685

[104] X. Feng, M. E. Bernard, T. Hunter, and Q. Chen, “Improving accuracy and robustness of deep convolutional neural network based thoracic OAR segmentation,” *Physics in Medicine & Biology*, vol. 65, no. 7, p. 07NT01, 2020. 10.1088/1361-6560/ab7877 PMC8035811 32079002

[105] T. Lustberg, J. van Soest, M. Gooding, D. Peressutti, P. Aljabar, J. van der Stoep, W. van Elmpt, and A. Dekker, “Clinical evaluation of atlas and deep learning based automatic contouring for lung cancer,” *Radiotherapy and Oncology*, vol. 126, no. 2, pp. 312–317, 2018. 10.1016/j.radonc.2017.11.012 29208513

[106] P. J. Doolan, S. Charalambous, Y. Roussakis, A. Leczynski, M. Peratikou, M. Benjamin, K. Ferentinos, I. Strouthos, C. Zamboglou, and E. Karagiannis, “A clinical evaluation of the performance of five commercial artificial intelligence contouring systems for radiotherapy,” *Frontiers in oncology*, vol. 13, p. 1213068, 2023. 10.3389/fonc.2023.1213068 PMC10436522 37601695

[107] C. McIntosh, M. Welch, A. McNiven, D. A. Jaffray, and T. G. Purdie, “Fully automated treatment planning for head and neck radiotherapy using a voxel-based dose prediction and dose mimicking method,” *Physics in Medicine & Biology*, vol. 62, no. 15, p. 5926, 2017. 10.1088/1361-6560/aa71f8 28486217

[108] S. Stathakis, G. Pissakas, A. Alexiou, B. Bertrand, P. Bondiau, L. Claude, T. Cuthbert, A. Damatopoulou, C. Dejean, C. Doukakis, et al., “Evaluation of AI vs. Clinical Experts SBRT-Thorax Computed Tomography OARs Delineation,” *International Journal of Radiation Oncology, Biology, Physics*, vol. 114, no. 3, pp. e102–e103, 2022.

[109] Y. Fu, Y. Lei, T. Wang, S. Tian, P. Patel, A. B. Jani, W. J. Curran, T. Liu, and X. Yang, “Pelvic multi-organ segmentation on cone-beam ct for prostate adaptive radiotherapy,” *Medical physics*, vol. 47, no. 8, pp. 3415–3422, 2020. 10.1002/mp.14196 PMC7429321 32323330

[110] J. Wasserthal, H.-C. Breit, M. T. Meyer, M. Pradella, D. Hinck, A. W. Sauter, T. Heye, D. T. Boll, J. Cyriac, S. Yang, et al., “Totalsegmentator: Robust segmentation of 104 anatomic structures in ct images,” *Radiology: Artificial Intelligence*, vol. 5, no. 5, 2023. 10.1148/ryai.230024 PMC10546353 37795137

[111] S. Strolin, M. Santoro, G. Paolani, I. Ammendolia, A. Arcelli, A. Benini, S. Bisello, R. Cardano, L. Cavallini, E. Deraco, et al., “How smart is artificial intelligence in organs delineation? Testing a CE and FDA-approved Deep-Learning tool using multiple expert contours delineated on planning CT images,” *Frontiers in Oncology*, vol. 13, p. 1089807, 2023. 10.3389/fonc.2023.1089807 PMC10019504 36937399

[112] V. Valentini, L. Boldrini, A. Damiani, and L. P. Muren, “Recommendations on how to establish evidence from auto-segmentation software in radiotherapy,” *Radiotherapy and Oncology*, vol. 112, no. 3, pp. 317–320, 2014. 10.1016/j.radonc.2014.09.014 25315862

[113] K. Men, H. Geng, T. Biswas, Z. Liao, and Y. Xiao, “Automated quality assurance of OAR contouring for lung cancer based on segmentation with deep active learning,” *Frontiers in Oncology*, vol. 10, p. 986, 2020. 10.3389/fonc.2020.00986 PMC7350536 32719742

[114] D. J. Rhee, C. E. Cardenas, H. Elhalawani, R. McCarroll, L. Zhang, J. Yang, A. S. Garden, C. B. Peterson, B. M. Beadle, and L. E. Court, “Automatic detection of contouring errors using convolutional neural networks,” *Medical physics*, vol. 46, no. 11, pp. 5086–5097, 2019. 10.1002/mp.13814 PMC6842055 31505046

[115] W. Gan, H. Wang, H. Gu, Y. Duan, Y. Shao, H. Chen, A. Feng, Y. Huang, X. Fu, Y. Ying, et al., “Automatic segmentation of lung tumors on CT images based on a 2D & 3D hybrid convolutional neural network,” *The British Journal of Radiology*, vol. 94, p. 20210038, 2021. 10.1259/bjr.20210038 PMC9328064 34347535

[116] S. P. Primakov, A. Ibrahim, J. E. van Timmeren, G. Wu, S. A. Keek, M. Beuque, R. W. Granzier, E. Lavrova, M. Scrivener, S. Sanduleanu, et al., “Automated detection and segmentation of non-small cell lung cancer computed tomography images,” *Nature communications*, vol. 13, no. 1, p. 3423, 2022. 10.1038/s41467-022-30841-3 PMC9198097 35701415

[117] S. Fischer, J. Kiechle, D. Lang, J. C. Peeken, and J. A. Schnabel, “Mask the Unknown: Assessing Different Strategies to Handle Weak Annotations in the MICCAI2023 Mediastinal Lymph Node Quantification Challenge,” 2024.

[118] S. S. Gay, C. E. Cardenas, C. Nguyen, T. J. Netherton, C. Yu, Y. Zhao, S. Skett, T. Patel, D. Adjogatse, T. Guerrero Urbano, et al., “Fully-automated, CT-only GTV contouring for palliative head and neck radiotherapy,” *Scientific reports*, vol. 13, no. 1, p. 21797, 2023. 10.1038/s41598-023-48944-2 PMC10709623 38066074

[119] W. Liao, J. He, X. Luo, M. Wu, Y. Shen, C. Li, J. Xiao, G. Wang, and N. Chen, “Automatic delineation of gross tumor volume based on magnetic resonance imaging by performing a novel semisupervised learning framework in nasopharyngeal carcinoma,” *International Journal of Radiation Oncology* Biology* Physics*, vol. 113, no. 4, pp. 893–902, 2022. 10.1016/j.ijrobp.2022.03.031 35381322

[120] J. A. Buchner, F. Kofler, L. Etzel, M. Mayinger, S. M. Christ, T. B. Brunner, A. Wittig, B. Menze, C. Zimmer, B. Meyer, et al., “Development and external validation of an mri-based neural network for brain metastasis segmentation in the aurora multicenter study,” *Radiotherapy and Oncology*, vol. 178, p. 109425, 2023. 10.1016/j.radonc.2022.11.014 36442609

[121] J. A. Buchner, J. C. Peeken, L. Etzel, I. Ezhov, M. Mayinger, S. M. Christ, T. B. Brunner, A. Wittig, B. H. Menze, C. Zimmer, et al., “Identifying core mri sequences for reliable automatic brain metastasis segmentation,” *Radiotherapy and Oncology*, vol. 188, p. 109901, 2023. 10.1016/j.radonc.2023.109901 37678623

[122] S. Tian, Y. Liu, X. Mao, X. Xu, C. Wang, G. Han, Y. Yang, J. Wang, S. He, and W. Zhang, “A multicenter study on deep learning for glioblastoma auto-segmentation with prior knowledge in multimodal imaging,” *International Journal of Radiation Oncology, Biology, Physics*, vol. 117, no. 2, p. e488, 2023.

[123] A.-L. Grosu, L. D. Sprague, and M. Molls, “Definition of target volume and organs at risk. Biological target volume,” *New Technologies in Radiation Oncology*, pp. 167–177, 2006.

[124] A. K. Berthelsen, J. Dobbs, E. Kjellén, T. Landberg, T. R. Möller, P. Nilsson, L. Specht, and A. Wambersie, “What’s new in target volume definition for radiologists in ICRU Report 71? How can the ICRU volume definitions be integrated in clinical practice?,” *Cancer Imaging*, vol. 7, no. 1, p. 104, 2007. 10.1102/1470-7330.2007.0013 PMC1906985 17594916

[125] K. Men, X. Chen, Y. Zhang, T. Zhang, J. Dai, J. Yi, and Y. Li, “Deep deconvolutional neural network for target segmentation of nasopharyngeal cancer in planning computed tomography images,” *Frontiers in oncology*, vol. 7, p. 315, 2017. 10.3389/fonc.2017.00315 PMC5770734 29376025

[126] A. R. Groendahl, I. S. Knudtsen, B. N. Huynh, M. Mulstad, Y. M. Moe, F. Knuth, O. Tomic, U. G. Indahl, T. Torheim, E. Dale, et al., “A comparison of methods for fully automatic segmentation of tumors and involved nodes in PET/CT of head and neck cancers,” *Physics in Medicine & Biology*, vol. 66, no. 6, p. 065012, 2021. 10.1088/1361-6560/abe553 33666176

[127] C. E. Cardenas, B. M. Beadle, A. S. Garden, H. D. Skinner, J. Yang, D. J. Rhee, R. E. McCarroll, T. J. Netherton, S. S. Gay, L. Zhang, et al., “Generating high-quality lymph node clinical target volumes for head and neck cancer radiation therapy using a fully automated deep learning-based approach,” *International Journal of Radiation Oncology* Biology* Physics*, vol. 109, no. 3, pp. 801–812, 2021. 10.1016/j.ijrobp.2020.10.005 PMC9472456 33068690

[128] D. Jin, D. Guo, T.-Y. Ho, A. P. Harrison, J. Xiao, C.-k. Tseng, and L. Lu, “Deep esophageal clinical target volume delineation using encoded 3D spatial context of tumors, lymph nodes, and organs at risk,” in *Medical Image Computing and Computer Assisted Intervention–MICCAI 2019: 22nd International Conference, Shenzhen, China, October 13–17, 2019, Proceedings, Part VI 22*, pp. 603–612, Springer, 2019.

[129] C. E. Cardenas, R. E. McCarroll, L. E. Court, B. A. Elgohari, H. Elhalawani, C. D. Fuller, M. J. Kamal, M. A. Meheissen, A. S. Mohamed, A. Rao, et al., “Deep learning algorithm for auto-delineation of high-risk oropharyngeal clinical target volumes with built-in dice similarity coefficient parameter optimization function,” *International Journal of Radiation Oncology* Biology* Physics*, vol. 101, no. 2, pp. 468–478, 2018. 10.1016/j.ijrobp.2018.01.114 PMC7473446 29559291

[130] Y. Xie, K. Kang, Y. Wang, M. J. Khandekar, H. Willers, F. K. Keane, and T. R. Bortfeld, “Automated clinical target volume delineation using deep 3D neural networks in radiation therapy of Non-small Cell Lung Cancer,” *Physics and Imaging in Radiation Oncology*, vol. 19, pp. 131–137, 2021. 10.1016/j.phro.2021.08.003 PMC8397906 34485718

[131] M. Kazemimoghadam, Z. Yang, M. Chen, A. Rahimi, N. Kim, P. Alluri, C. Nwachukwu, W. Lu, and X. Gu, “A deep learning approach for automatic delineation of clinical target volume in stereotactic partial breast irradiation (S-PBI),” *Physics in Medicine & Biology*, vol. 68, no. 10, p. 105011, 2023. 10.1088/1361-6560/accf5e PMC10325028 37084739

[132] G. Dipasquale, X. Wang, V. Chatelain-Fontanella, V. Vinh-Hung, and R. Miralbell, “Automatic segmentation of breast in prone position: correlation of similarity indexes and breast pendulousness with dose/volume parameters,” *Radiotherapy and Oncology*, vol. 120, no. 1, pp. 124–127, 2016. 10.1016/j.radonc.2016.04.041 27178144

[133] P. Buelens, S. Willems, L. Vandewinckele, W. Crijns, F. Maes, and C. Weltens, “Clinical evaluation of a deep learning model for segmentation of target volumes in breast cancer radiotherapy,” *Radiotherapy and Oncology*, vol. 171, pp. 84–90, 2022. 10.1016/j.radonc.2022.04.015 35447286

[134] J. Shi, X. Ding, X. Liu, Y. Li, W. Liang, and J. Wu, “Automatic clinical target volume delineation for cervical cancer in CT images using deep learning,” *Medical Physics*, vol. 48, no. 7, pp. 3968–3981, 2021. 10.1002/mp.14898 33905545

[135] Y. Chang, Z. Wang, Z. Peng, J. Zhou, Y. Pi, X. G. Xu, and X. Pei, “Clinical application and improvement of a CNN-based autosegmentation model for clinical target volumes in cervical cancer radiotherapy,” *Journal of Applied Clinical Medical Physics*, vol. 22, no. 11, pp. 115–125, 2021. 10.1002/acm2.13440 PMC8598149 34643320

[136] Z. Liu, X. Liu, H. Guan, H. Zhen, Y. Sun, Q. Chen, Y. Chen, S. Wang, and J. Qiu, “Development and validation of a deep learning algorithm for auto-delineation of clinical target volume and organs at risk in cervical cancer radiotherapy,” *Radiotherapy and Oncology*, vol. 153, pp. 172–179, 2020. 10.1016/j.radonc.2020.09.060 33039424

[137] Z. Liu, W. Chen, H. Guan, H. Zhen, J. Shen, X. Liu, A. Liu, R. Li, J. Geng, J. You, et al., “An adversarial deep-learning-based model for cervical cancer CTV segmentation with multicenter blinded randomized controlled validation,” *Frontiers in Oncology*, vol. 11, p. 702270, 2021. 10.3389/fonc.2021.702270 PMC8417437 34490103

[138] N. Shusharina, J. Söderberg, D. Edmunds, F. Löfman, H. Shih, and T. Bortfeld, “Automated delineation of the clinical target volume using anatomically constrained 3D expansion of the gross tumor volume,” *Radiotherapy and Oncology*, vol. 146, pp. 37–43, 2020. 10.1016/j.radonc.2020.01.028 PMC10660950 32114264

[139] N. Aldoj, F. Biavati, F. Michallek, S. Stober, and M. Dewey, “Automatic prostate and prostate zones segmentation of magnetic resonance images using DenseNet-like U‑net,” *Scientific reports*, vol. 10, no. 1, p. 14315, 2020. 10.1038/s41598-020-71080-0 PMC7459118 32868836

[140] C.-Y. Ma, J.-Y. Zhou, X.-T. Xu, J. Guo, M.-F. Han, Y.-Z. Gao, H. Du, J. N. Stahl, and J. S. Maltz, “Deep learning-based auto-segmentation of clinical target volumes for radiotherapy treatment of cervical cancer,” *Journal of Applied Clinical Medical Physics*, vol. 23, no. 2, p. e13470, 2022. 10.1002/acm2.13470 PMC8833283 34807501

[141] N. Bi, J. Wang, T. Zhang, X. Chen, W. Xia, J. Miao, K. Xu, L. Wu, Q. Fan, L. Wang, et al., “Deep learning improved clinical target volume contouring quality and efficiency for postoperative radiation therapy in non-small cell lung cancer,” *Frontiers in oncology*, vol. 9, p. 1192, 2019. 10.3389/fonc.2019.01192 PMC6863957 31799181

[142] X. Zhao, P. Xie, M. Wang, W. Li, P. J. Pickhardt, W. Xia, F. Xiong, R. Zhang, Y. Xie, J. Jian, et al., “Deep learning–based fully automated detection and segmentation of lymph nodes on multiparametric-mri for rectal cancer: A multicentre study,” *EBioMedicine*, vol. 56, 2020. 10.1016/j.ebiom.2020.102780 PMC7276514 32512507

[143] X. Liu, K.-W. Li, R. Yang, and L.-S. Geng, “Review of Deep Learning Based Automatic Segmentation for Lung Cancer Radiotherapy,” *Frontiers in Oncology*, vol. 11, 2021. 10.3389/fonc.2021.717039 PMC8323481 34336704

[144] D. Huang, H. Bai, L. Wang, Y. Hou, L. Li, Y. Xia, Z. Yan, W. Chen, L. Chang, and W. Li, “The Application and Development of Deep Learning in Radiotherapy: A Systematic Review,” *Technology in Cancer Research & Treatment*, vol. 20, p. 15330338211016386, 2021. 10.1177/15330338211016386 PMC8216350 34142614

[145] C. Robert, A. Munoz, D. Moreau, J. Mazurier, G. Sidorski, A. Gasnier, G. Beldjoudi, V. Grégoire, E. Deutsch, P. Meyer, and L. Simon, “Clinical implementation of deep-learning based auto-contouring tools-Experience of three French radiotherapy centers,” *Cancer Radiotherapie: Journal De La Societe Francaise De Radiotherapie Oncologique*, vol. 25, pp. 607–616, Oct. 2021. 10.1016/j.canrad.2021.06.023 34389243

[146] T. J. Netherton, C. E. Cardenas, D. J. Rhee, L. E. Court, and B. M. Beadle, “The Emergence of Artificial Intelligence within Radiation Oncology Treatment Planning,” *Oncology*, vol. 99, no. 2, pp. 124–134, 2021. 10.1159/000512172 33352552

[147] J. K. Udupa, T. Liu, C. Jin, L. Zhao, D. Odhner, Y. Tong, V. Agrawal, G. Pednekar, S. Nag, T. Kotia, et al., “Combining natural and artificial intelligence for robust automatic anatomy segmentation: Application in neck and thorax auto-contouring,” *Medical physics*, vol. 49, no. 11, pp. 7118–7149, 2022. 10.1002/mp.15854 PMC10087050 35833287

[148] J. Wong, A. Fong, N. McVicar, S. Smith, J. Giambattista, D. Wells, C. Kolbeck, J. Giambattista, L. Gondara, and A. Alexander, “Comparing deep learning-based auto-segmentation of organs at risk and clinical target volumes to expert inter-observer variability in radiotherapy planning,” *Radiotherapy and Oncology*, vol. 144, pp. 152–158, 2020. 10.1016/j.radonc.2019.10.019 31812930

[149] C. Fiorino, M. Reni, A. Bolognesi, G. M. Cattaneo, and R. Calandrino, “Intra-and inter-observer variability in contouring prostate and seminal vesicles: implications for conformal treatment planning,” *Radiotherapy and oncology*, vol. 47, no. 3, pp. 285–292, 1998. 10.1016/s0167-8140(98)00021-8 9681892

[150] L. Caravatta, G. Macchia, G. C. Mattiucci, A. Sainato, N. L. Cernusco, G. Mantello, M. Di Tommaso, M. Trignani, A. De Paoli, G. Boz, et al., “Inter-observer variability of clinical target volume delineation in radiotherapy treatment of pancreatic cancer: a multi-institutional contouring experience,” *Radiation oncology*, vol. 9, pp. 1–9, 2014. 10.1186/1748-717X-9-198 PMC4261525 25199768

[151] M. Altman, J. Kavanaugh, H. Wooten, O. Green, T. DeWees, H. Gay, W. Thorstad, H. Li, and S. Mutic, “A framework for automated contour quality assurance in radiation therapy including adaptive techniques,” *Physics in Medicine & Biology*, vol. 60, no. 13, p. 5199, 2015. 10.1088/0031-9155/60/13/5199 26083863

[152] M. Claessens, V. Vanreusel, G. De Kerf, I. Mollaert, F. Löfman, M. J. Gooding, C. Brouwer, P. Dirix, and D. Verellen, “Machine learning-based detection of aberrant deep learning segmentations of target and organs at risk for prostate radiotherapy using a secondary segmentation algorithm,” *Physics in Medicine & Biology*, vol. 67, no. 11, p. 115014, 2022. 10.1088/1361-6560/ac6fad 35561701

[153] X. Chen, K. Men, B. Chen, Y. Tang, T. Zhang, S. Wang, Y. Li, and J. Dai, “CNN-based quality assurance for automatic segmentation of breast cancer in radiotherapy,” *Frontiers in Oncology*, vol. 10, p. 524, 2020. 10.3389/fonc.2020.00524 PMC7212344 32426272

[154] I. Ezhov, K. Scibilia, K. Franitza, F. Steinbauer, S. Shit, L. Zimmer, J. Lipkova, F. Kofler, J. C. Paetzold, L. Canalini, et al., “Learn-morph-infer: a new way of solving the inverse problem for brain tumor modeling,” *Medical Image Analysis*, vol. 83, p. 102672, 2023. 10.1016/j.media.2022.102672 36395623

[155] J. Lipková, P. Angelikopoulos, S. Wu, E. Alberts, B. Wiestler, C. Diehl, C. Preibisch, T. Pyka, S. E. Combs, P. Hadjidoukas, et al., “Personalized radiotherapy design for glioblastoma: Integrating mathematical tumor models, multimodal scans, and bayesian inference,” *IEEE transactions on medical imaging*, vol. 38, no. 8, pp. 1875–1884, 2019. 10.1109/TMI.2019.2902044 PMC7170051 30835219

[156] M. Balcerak, I. Ezhov, P. Karnakov, S. Litvinov, P. Koumoutsakos, J. Weidner, R. Z. Zhang, J. S. Lowengrub, B. Wiestler, and B. Menze, “Individualizing glioma radiotherapy planning by optimization of a data and physics informed discrete loss,” *arXiv preprint arXiv:2312.05063*, 2023. 10.1038/s41467-025-60366-4 PMC12219029 40593490

[157] M.-C. Metz, I. Ezhov, L. Zimmer, J. C. Peeken, J. A. Buchner, J. Lipkova, F. Kofler, D. Waldmannstetter, C. Delbridge, C. Diehl, et al., “Towards image-based personalization of glioblastoma therapy a clinical and biological validation study of a novel, deep learning-driven tumor growth model,” 2023. 10.1093/noajnl/vdad171 PMC10907005 38435962

[158] M. Lê, H. Delingette, J. Kalpathy-Cramer, E. R. Gerstner, T. Batchelor, J. Unkelbach, and N. Ayache, “Personalized radiotherapy planning based on a computational tumor growth model,” *IEEE transactions on medical imaging*, vol. 36, no. 3, pp. 815–825, 2016. 10.1109/TMI.2016.2626443 28113925

[159] F. Dittmann, B. Menze, E. Konukoglu, and J. Unkelbach, “Use of diffusion tensor images in glioma growth modeling for radiotherapy target delineation,” in *Multimodal Brain Image Analysis: Third International Workshop, MBIA 2013, Held in Conjunction with MICCAI 2013, Nagoya, Japan, September 22, 2013, Proceedings 3*, pp. 63–73, Springer, 2013.

[160] M. B. Jensen, T. L. Guldberg, A. Harbøll, S. Lukacova, and J. F. Kallehauge, “Diffusion tensor magnetic resonance imaging driven growth modeling for radiotherapy target definition in glioblastoma,” *Acta Oncologica*, vol. 56, no. 11, pp. 1639–1643, 2017. 10.1080/0284186X.2017.1374559 28893125

[161] O. Rouvière, T. Vitry, and D. Lyonnet, “Imaging of prostate cancer local recurrences: why and how?,” *European radiology*, vol. 20, pp. 1254–1266, 2010. 10.1007/s00330-009-1647-4 19921202

[162] T. Dong, C. Yang, B. Cui, T. Zhang, X. Sun, K. Song, L. Wang, B. Kong, and X. Yang, “Development and validation of a deep learning radiomics model predicting lymph node status in operable cervical cancer,” *Frontiers in Oncology*, vol. 10, p. 464, 2020. 10.3389/fonc.2020.00464 PMC7179686 32373511

[163] D. Kong, W. Shan, Y. Zhu, Q. Xu, S. Duan, and L. Guo, “Preliminary study on ct contrast-enhanced radiomics for predicting central cervical lymph node status in patients with thyroid nodules,” *Frontiers in Oncology*, vol. 13, p. 1060674, 2023. 10.3389/fonc.2023.1060674 PMC9935823 36816945

[164] T. Haraguchi, Y. Kobayashi, D. Hirahara, T. Kobayashi, E. Takaya, M. T. Nagai, H. Tomita, J. Okamoto, Y. Kanemaki, and K. Tsugawa, “Radiomics model of diffusion-weighted whole-body imaging with background signal suppression (dwibs) for predicting axillary lymph node status in breast cancer,” *Journal of X‑Ray Science and Technology*, no. Preprint, pp. 1–14, 2023. 10.3233/XST-230009 37038802

[165] J. M. Rogasch, L. Michaels, G. L. Baumgärtner, N. Frost, J.-C. Rückert, J. Neudecker, S. Ochsenreither, M. Gerhold, B. Schmidt, P. Schneider, et al., “A machine learning tool to improve prediction of mediastinal lymph node metastases in non-small cell lung cancer using routinely obtainable [18f] fdg-pet/ct parameters,” *European Journal of Nuclear Medicine and Molecular Imaging*, pp. 1–12, 2023. 10.1007/s00259-023-06145-z PMC10199849 36820890

[166] P. Flechsig, P. Frank, C. Kratochwil, G. Antoch, D. Rath, J. Moltz, M. Rieser, A. Warth, H.-U. Kauczor, L. H. Schwartz, et al., “Radiomic analysis using density threshold for fdg-pet/ct-based n‑staging in lung cancer patients,” *Molecular imaging and biology*, vol. 19, pp. 315–322, 2017. 10.1007/s11307-016-0996-z 27539308

[167] J. C. Peeken, M. A. Shouman, M. Kroenke, I. Rauscher, T. Maurer, J. E. Gschwend, M. Eiber, and S. E. Combs, “A ct-based radiomics model to detect prostate cancer lymph node metastases in psma radioguided surgery patients,” *European Journal of Nuclear Medicine and Molecular Imaging*, vol. 47, pp. 2968–2977, 2020. 10.1007/s00259-020-04864-1 PMC7680305 32468251

[168] H. Tomita, T. Yamashiro, J. Heianna, T. Nakasone, Y. Kimura, H. Mimura, and S. Murayama, “Nodal-based radiomics analysis for identifying cervical lymph node metastasis at levels i and ii in patients with oral squamous cell carcinoma using contrast-enhanced computed tomography,” *European Radiology*, pp. 1–10, 2021. 10.1007/s00330-021-07758-4 33787970

[169] A. Hartenstein, F. Lübbe, A. D. Baur, M. M. Rudolph, C. Furth, W. Brenner, H. Amthauer, B. Hamm, M. Makowski, and T. Penzkofer, “Prostate cancer nodal staging: using deep learning to predict 68ga-psma-positivity from ct imaging alone,” *Scientific reports*, vol. 10, no. 1, p. 3398, 2020. 10.1038/s41598-020-60311-z PMC7042227 32099001

[170] J. C. Peeken, M. Molina-Romero, C. Diehl, B. H. Menze, C. Straube, B. Meyer, C. Zimmer, B. Wiestler, and S. E. Combs, “Deep learning derived tumor infiltration maps for personalized target definition in Glioblastoma radiotherapy,” *Radiotherapy and Oncology*, vol. 138, pp. 166–172, 2019. 10.1016/j.radonc.2019.06.031 31302391

[171] S. Rathore, H. Akbari, J. Doshi, G. Shukla, M. Rozycki, M. Bilello, R. Lustig, and C. Davatzikos, “Radiomic signature of infiltration in peritumoral edema predicts subsequent recurrence in glioblastoma: implications for personalized radiotherapy planning,” *Journal of Medical Imaging*, vol. 5, no. 2, pp. 021219–021219, 2018. 10.1117/1.JMI.5.2.021219 PMC5831697 29531967

[172] X. Xia, J. Wang, Y. Li, J. Peng, J. Fan, J. Zhang, J. Wan, Y. Fang, Z. Zhang, and W. Hu, “An artificial intelligence-based full-process solution for radiotherapy: a proof of concept study on rectal cancer,” *Frontiers in Oncology*, vol. 10, p. 616721, 2021. 10.3389/fonc.2020.616721 PMC7886996 33614500

